# Sampling impacts the assessment of tooth growth and replacement rates in archosaurs: implications for paleontological studies

**DOI:** 10.7717/peerj.9918

**Published:** 2020-09-18

**Authors:** Jens C.D. Kosch, Lindsay E. Zanno

**Affiliations:** 1 Paleontology, North Carolina Museum of Natural Sciences, Raleigh, NC, USA; 2 Department of Biological Sciences, North Carolina State University, Raleigh, NC, USA

**Keywords:** Tooth formation, Tooth replacement, Archosauria, Sampling effects, Alligator, von Ebner lines, Error bars

## Abstract

Dietary habits in extinct species cannot be directly observed; thus, in the absence of extraordinary evidence, they must be reconstructed with a combination of morphological proxies. Such proxies often include information on dental organization and function such as tooth formation time and tooth replacement rate. In extinct organisms, tooth formation times and tooth replacement rate are calculated, in part via extrapolation of the space between incremental lines in dental tissues representing daily growth (von Ebner Line Increment Width; VEIW). However, to date, little work has been conducted testing assumptions about the primary data underpinning these calculations, specifically, the potential impact of differential sampling and data extrapolation protocols. To address this, we tested a variety of intradental, intramandibular, and ontogentic sampling effects on calculations of mean VEIW, tooth formation times, and replacement rates using histological sections and CT reconstructions of a growth series of three specimens of the extant archosaurian *Alligator mississippiensis*. We find transect position within the tooth and transect orientation with respect to von Ebner lines to have the greatest impact on calculations of mean VEIW—a maximum number of VEIW measurements should be made as near to the central axis (CA) as possible. Measuring in regions away from the central axis can reduce mean VEIW by up to 36%, causing inflated calculations of tooth formation time. We find little demonstrable impact to calculations of mean VEIW from the practice of subsampling along a transect, or from using mean VEIW derived from one portion of the dentition to extrapolate for other regions of the dentition. Subsampling along transects contributes only minor variations in mean VEIW (<12%) that are dwarfed by the standard deviation (SD). Moreover, variation in VEIW with distance from the pulp cavity likely reflects idiosyncratic patterns related to life history, which are difficult to control for; however, we recommend increasing the number of VEIW measured to minimize this effect. Our data reveal only a weak correlation between mean VEIW and body length, suggesting minimal ontogenetic impacts. Finally, we provide a relative SD of mean VEIW for Alligator of 29.94%, which can be used by researchers to create data-driven error bars for tooth formation times and replacement rates in fossil taxa with small sample sizes. We caution that small differences in mean VEIW calculations resulting from non-standardized sampling protocols, especially in a comparative context, will produce inflated error in tooth formation time estimations that intensify with crown height. The same holds true for applications of our relative SD to calculations of tooth formation time in extinct taxa, which produce highly variable maximum and minimum estimates in large-toothed taxa (e.g., 718–1,331 days in *Tyrannosaurus*).

## Introduction

Tooth replacement rate provides key information on the function and evolution of the dentition (Edmund, 1960; Osborn, 1970, 1975; Richman, Whitlock & Abramyan, 2013; Whitlock & Richman, 2013; Schwarz et al., 2015; LeBlanc et al., 2016; Bramble et al., 2017). Such data can be used to infer aspects of the paleobiology of extinct taxa including metabolic activity/investment, dietary preferences, and behavior (Johnston, 1979; Barrett, 2014; Salakka, 2014; D’Emic et al., 2013, 2019; D’Emic & Carrano, 2019; Brink et al., 2015; Button et al., 2017; D’Emic et al., 2019). However, tooth growth and exfoliation cannot be directly observed in extinct species, therefore tooth replacement rate must be estimated via growth lines preserved within dentin (von Ebner Lines) and/or incremental growth lines of enamel that record different rhythms (see Smith (2004) for a review). Within living crocodilians, von Ebner lines are known to represent daily dentin deposition (Erickson, 1992, 1996a). Paleontological studies have therefore used direct von Ebner line counts, or estimates derived by measuring dentin thickness divided by the mean width between von Ebner lines (so called “von Ebner Line Increment Width”, VEIW), to estimate tooth formation times and replacement rates in extinct species (Erickson, 1996b; Sereno et al., 2007; D’Emic et al., 2013, 2019; Garcia & Zurriaguz, 2016; Erickson et al., 2017; Ricart et al., 2019). This practice makes the accurate estimation of von Ebner line counts and mean VEIW a critical consideration, as errors in the calculation of either will have cascading effects, ultimately resulting in erroneous paleobiological inferences and macroevolutionary trends.

The challenges of working with fossil data constrain sampling approaches for deriving von Ebner line count and mean VEIW in extinct species. For example, because a researcher cannot always choose the location or orientation for consumptive sampling, they often have to calculate mean VEIW from one region of the tooth and apply these data to different transect locations within the same tooth (Erickson, 1992, 1996b; Sereno et al., 2007; Gren, 2011; D’Emic et al., 2013, 2019; Gren & Lindgren, 2013; Garcia & Zurriaguz, 2016; Kear et al., 2017; Ricart et al., 2019). Likewise, researchers may have to calculate mean VIEW from one tooth position within the jaw and then apply these values to other tooth positions (Kosch, 2014; Schwarz et al., 2015; Garcia & Zurriaguz, 2016; D’Emic & Carrano, 2019; D’Emic et al., 2019). Moreover, because von Ebner lines are not always visible along the entire transect, mean VEIWs are typically derived from a transect subsample, as opposed to being calculated from the entire transect length (Erickson, 1992, 1996a, 1996b; Gren, 2011; Gren & Lindgren, 2013; Button et al., 2017; Erickson et al., 2017; Kear et al., 2017; Ricart et al., 2019; D’Emic et al., 2019). These practices rely on a series of data extrapolations that can introduce possible intradental and intramandibular sampling effects.

Intradental data extrapolations are often based on one or multiple of the following assumptions: (1) mean VEIW is constant regardless of the developmental age of the tooth (i.e., does not vary significantly or consistently with distance from the pulp cavity); (2) the maximum number of von Ebner lines are preserved at any transect location (i.e., a transect taken anywhere on the tooth from pulp cavity to crown will capture all von Ebner lines reflecting the maximum age of the tooth); and (3) mean VEIW is consistent regardless of the transect position used for sampling (i.e., does not vary across the tooth). Assumption 1 forms the basis for using a subsection of a transect to derive a mean VEIW for a tooth (Erickson, 1992, 1996a, 1996b; Gren, 2011; Gren & Lindgren, 2013; Erickson et al., 2017; Kear et al., 2017; Ricart et al., 2019; D’Emic et al., 2019), but contrasts with a competing hypothesis that teeth have different growth rates during their formation, which would result in wider VEIWs depending on distance from the pulp cavity (Y-H. Wu, 2018, personal communication, Lawson, Wake & Beck, 1971; Hanai & Tsuihiji, 2018; Finger, Thomson & Isberg, 2019), as well as with observations of flexible replacement rates coupled with metabolic activity (often seasonally influenced) in extant reptiles (Cooper (1966) in *Anguis fragilis*, Delgado, Davit-Beal & Sire (2003) in *Chalcides sexlineatus* and *Chalcides viridanus*) and mammals (Klevezal, 1996: 66f). Assumption 2 forms the basis for the use of transverse sections to derive tooth formation times (Sereno et al., 2007; Gren, 2011; Gren & Lindgren, 2013; Kear et al., 2017; Ricart et al., 2019). This assumption was criticized by D’Emic et al. (2013) on grounds that not all von Ebner lines present in a tooth are exposed in transverse section. Assumption three forms the basis for approaches that measure VEIWs and calculate a mean VEIW based on transects that stretch from the tooth’s center near the pulp cavity to the marginal parts of the tooth, either in transverse sections of the tooth (Gren, 2011; Gren & Lindgren, 2013; Kear et al., 2017; Ricart et al., 2019) or multiple groups of measurements in a longitudinal section (“zig-zag pattern”, Erickson, 1992, 1996a, 1996b; D’Emic et al., 2013, 2019 (in part)).

Not only is it difficult to mitigate intradental sampling effects, one cannot generally sample all teeth in a tooth row; therefore, many studies rely on the assumption that VEIW is constant across the tooth row and between upper and lower arcades. For example, previous studies of fossil archosaurs utilizing von Ebner line counts and/or mean VEIW to derive tooth formation times and replacement rates, base these estimates on teeth within a single respective tooth family within the dentition (either mesial teeth (Erickson, 1992, 1996a; Sereno et al., 2007; D’Emic et al., 2013), unspecified “mean-sized teeth” (Erickson, 1996b; Erickson et al., 2017), molariform distal teeth (Ricart et al., 2019), or isolated teeth of uncertain position within the jaw (Garcia & Zurriaguz, 2016; D’Emic et al., 2019)). The use of “mean sized teeth” (Erickson, 1992, 1996a, 1996b) relies on the assumption that mean VEIW does not differ systematically according to tooth position and thus is constant in all tooth positions. D’Emic et al. (2013) derived a mean VEIW from premaxillary teeth in sauropods; however, subsequent studies applied transfer functions for tooth length to whole dentitions to derive tooth formation times in sauropods (Schwarz et al., 2015; Kosch, 2014; D’Emic et al., 2019). In a similar fashion, D’Emic et al. (2019) applied mean VEIW derived from isolated teeth from various mandibular elements of theropods to other mandibular elements to estimate tooth formation times and replacement rates. These practices have the potential to introduce intramandibular sampling effects. Finally, Erickson (1992, 1996a, 1996b) concluded that VEIW changes during ontogeny, with wider VEIWs characterizing larger individuals. Such differences could introduce ontogenetic sampling effects.

Here we explore the impact of intradental, intramandibular, and ontogenetic sampling effects on calculations of mean VIEW, tooth formation times and replacement rates by sampling multiple teeth along the tooth row of different ontogenetic stages of the extant archosaurian taxon *Alligator mississippiensis*. We also explore the distribution of VEIW within our sample and apply standard deviations (SD) derived from an extant archosaur to existing calculations of tooth formation time in fossil vertebrates, in order to assess the potential impact of VEIW variation. The results serve to inform best practices for sampling in studies of dental organization and begin to quantify potential error in tooth growth and replacement rate estimates in extinct taxa.

## Materials and Methods

### Specimens and sampling

**Figure 1:**
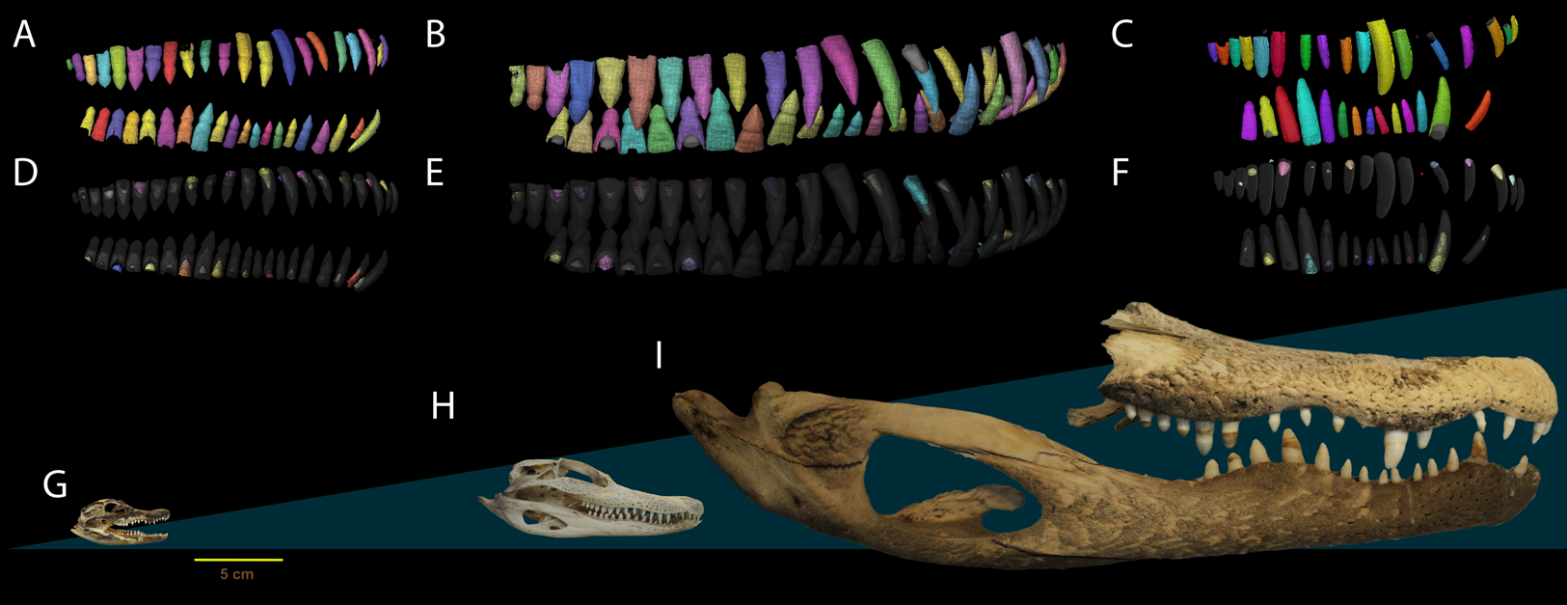
Dental anatomy of Alligator used in this study. (A, D, G) NCSM 100803; (B, E, H) NCSM 100804 and; (C, F, I) NCSM 100805. Segmented dentition showing (A–C) functional teeth in color and (D–F) replacement teeth in color. Replacement teeth and functional tooth of each position are presented in different shades of the same color. Segmented dentition not to scale, skulls (G–I) to scale. Scale bar 5 cm.

To assess ontogenetic sampling effects, we examined the dentition across an ontogenetic series of three individuals of *Alligator mississippiensis*. We estimated body length of our two smallest specimens using the regressions of Farlow et al. (2005) (femur length against body length). Our smallest individual (NCSM 100803) has an estimated body length of 37.1 cm (femur length = 24.55 mm), which corresponds to the size expected of young yearling (larger than a hatchling but 33% smaller than a yearling at the end of its first year) (Khan & Tansel, 2000) (Figs. 1A, 1D and 1G) and our medium sized individual (NCSM 100804) has an estimated body length of 90.6 cm (femur length = 61.58 mm), corresponding to the upper end of the size range expected of a 2 year old (Khan & Tansel, 2000) (Figs. 1B, 1E and 1H). There was no associated femur with our largest specimen (NCSM 100805), therefore we used two methods to approximate an estimated body length range (Figs. 1C, 1F and 1I). We used mandible length as a proxy for skull length, which we then used to estimate total length from the regressions in Farlow et al. (2005). We also used mandible length to estimate Snout-Vent length, and then derive total body length using regression equations derived by Wu et al. (2006) for the congener *A. sinensis*. These produced an estimated body length of between 3.6 and 3.9 m, which corresponds to an age of more than 16 years according to Khan & Tansel (2000). No life history data were available for specimens, the estimated ages correspond to the size range given for age categories in Table 3 of Khan & Tansel (2000), following Neill (1971).

All material was scanned at Duke University in a micro-CT Nikon XTH 225 ST. CT images were taken with a setting of 170 kV and 86 mA and slice thickness of 31.7 µm for the small *Alligator*. The skull of the medium sized *Alligator* was scanned in two scan processes, one with 135 kV and154 mA and one with 130 kV and 170 mA, both with a slice thickness of 45.2 µm. For the large *Alligator*, the upper tooth row was scanned with 150 kV and 134 to 139 mA and the lower tooth row with 150 kV to 139 to 144 mA using a slice thickness of 61.3 µm. All files were saved in .tif format. Data reconstruction was done with a RECO1 workstation. Composite files for the entire skulls or complete elements (upper and lower tooth rows of the large *Alligator*) were constructed in AVIZO 9.0. Those files were used for two different purposes: an initial inspection that aided in the decision which alveoli should be sampled for histological sections of teeth and surrounding hard tissue, and to create digital models of the teeth in order to ascertain measurements of functional and replacement teeth of the quadrant of the jaw from which histological samples would be taken. Foremost among these measurements was central axis height (CAH), the distance between the tip of the pulp cavity and the crown apex, which equals the sum of all VEIWs measured along the central axis (plus apical enamel thickness). The combination of resolution and contrast of the X-ray micro-CT scans does not allow for the clear distinction of the enamel dentin junction in all teeth (Jones et al., 2018).

We chose 12 tooth positions that exhibited both a functional tooth and replacement teeth for sampling (Pmx 1, Pmx3, Mx 6, Mx 7, Mx 12, Mx 13, Dent 2, Dent 4, Dent 11, Dent 12, Dent 18, Dent 19). The distalmost teeth of the largest specimen were not preserved, therefore we approximated these tooth positions by sampling the 15th and 16th alveolus. Sampling of multiple tooth positions across the length of each dentary helped to access intramandibular sampling effects. Some of our data collection required sampling of the same position across multiple individuals. In these cases, we chose a subset of tooth positions that possessed at least one functional tooth and at least one replacement tooth (mesial dentition only as distal dentition often lacked replacement teeth) and also represented a widespread distribution across the tooth row and dentigerous elements (mesial premaxillary teeth, mesial and distal maxillary teeth, and their counterparts in the dentary) across all three individuals.

### Histology

**Figure 2:**
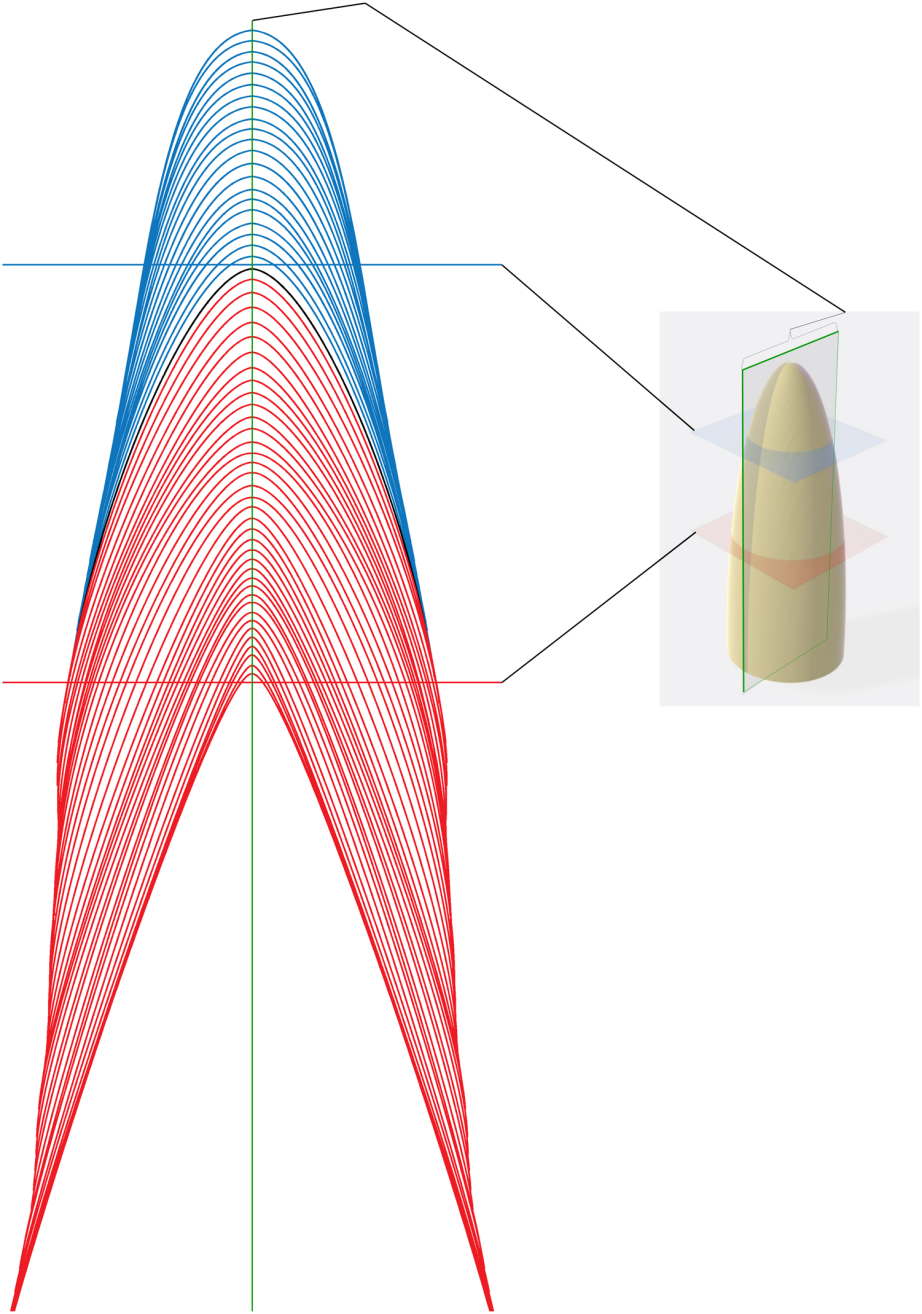
Schematic representation of section planes and transects. Schematic representation of different sectioning planes with examples of raw data for different transect locations. Blue von Ebner lines (VEL) appear in transverse section taken in the blue plane. Red VEL appear in a transverse section in the red plane. A section in the plane of the central axis (green with brackets) will cross all VELs.

Histological slides were prepared following standard thin-sectioning protocols (Lamm, 2013). First, the respective areas of interest were separated using a diamond bladed ISOMET 1000 precision saw. The segments were prepared for embedding by dehydration in progressive ethanol baths (70%, 90%, and 100%) and defatted in acetone. Samples were not demineralized, which is known to cause shrinkage (Dean, 1998; Smith, 2004) and can impact tooth measurements. Next, the samples were embedded in Epo-Tek 301 Epoxy Resin. No staining was performed. The embedded jaw elements were cut close to the intended plane of the section (Fig. 2) and ground using silica carbide paper (120–1,200 grit) before being fixed to a slide, cut again and ground down to 0.13–0.56 mm thickness. The sections were studied with a Nikon Eclipse Ci POL microscope equipped with a polarizer and a lambda filter, and photographed using an iPhone 7 camera or a Rollei Powerflex 470 camera.

#### Identification of daily growth lines

We observed incremental growth lines ranging 5–39 µm within the sample and 6–38 µm within a single tooth. We found no smaller incremental lines even between the von Ebner line with the widest distance. As the incremental lines observed by us conform to the width of daily deposited von Ebner lines reported from other archosaurs (Allison et al., 2019; D’Emic et al., 2013, 2019; Erickson, 1992, 1996a, 1996b; Gren & Lindgren, 2013; Ricart et al., 2019; Sokolskyi, Zanno & Kosch, 2019) we conclude that we did not observe intradian (Smith, 2004) or ultradian (Ohtsuka & Shinoda, 1995; Klevezal, 1996) lines that might occur as bands subdividing daily lines. The range of incremental line width observed in our sample also falls in similar range to the VEIW observed in the ever-growing incisors of rodents (Rosenberg & Simmons, 1980; Ohtsuka & Shinoda, 1995; Klevezal, 1996; Smith, 2004). Under the assumption that the observed incremental lines are longer period Andresen lines the formation times and replacement rates become unrealistically long exceeding observed exfoliation rates (Erickson, 1996a) by 7–13 times. The presumed daily growth lines also are more similar to the short period growth lines observed in mosasaur teeth that also sow longer period Andresen lines (Gren & Lindgren, 2013). Therefore, we interpret all our von Ebner line as daily incremental growth lines.

#### Von Ebner line count and measurements

Photographs were either stitched together into a composite within Adobe Photoshop 2015 or directly opened in Image J, whereupon a transect line was applied and the sections of the von Ebner lines crossing the transect were marked with an arrow symbol. Increment widths were measured using the Image J inbuilt measurement tool and measurements were exported to Excel 2015 to compute averages. Minimum and maximum VEIW in a given transect were used in combination with the transect length and the CAH to compute absolute maximum and minimum tooth ages represented by the measured transect and the tooth respectively. Upper and lower limits of tooth formation time were computed by subtracting (for maximum ages) and adding (for minimum ages) the SD of the VEIW from the mean VEIW and applying these values to the transect length and the CAH. This strategy of measuring VEIWs allowed us to test the assumptions that data derived from one tooth position can reliably be applied to a tooth from a different tooth position and that mean VEIW does not vary significantly or consistently with distance from the pulp cavity.

Hierarchically there are three different mean VEIWs used in different steps. The mean VEIW of a transect, as described above; the mean VEIW of all transects of a tooth, which was used in calculating the tooth formation time used to determine replacement rate; the mean VEIW of a specimen was used to compare VEIW through ontogeny and with other datasets. Tooth height and CAH were measured in AVIZO 9.0 (Fig. 1).

In the large *Alligator* some tooth crowns were damaged, with small chips of the apices missing and cracks running through parts of the crown. Some also showed a considerable degree of tooth wear. In these cases, the crowns were digitally reconstructed to their full height in Avizo. Cracks and missing chips were digitally infilled with voxels assigned as reconstructed dentin up to a point where tooth wear was visible if signs of wear could be identified. Further reconstruction followed the curvature of the crown’s shape, covering every exposed dentin surface with a layer at least two voxels deep that were assigned to a different material that represents dentin and enamel lost in tooth wear. These reconstructed crowns were used when measuring CAH and tooth height for these teeth. The original values without reconstruction are presented in the Data S1.

Our raw measurement of CAH and tooth height include both enamel and dentin thickness. The enamel layer in extant crocodylians is thin (Enax et al., 2013) and scales nearly isometrically (Schmiegelow, Sellers & Holliday, 2016; Sellers, Schmiegelow & Holliday, 2019), with minor changes related to tooth shape (intrafamilial heterodonty) (Osborn, 1975; Kieser et al., 1993; Gignac, 2010; D’Amore et al., 2019) as opposed to size. To calculate CAH as a measure of dentin only, we subtracted the average enamel thickness value, and used this corrected CAH value in our calculations. The average enamel thickness was derived from measurements perpendicular to the enamel dentin junction taken from photographs of thin sections of representative teeth using the measurement tool in ImageJ. For the small and medium sized specimens three photographs with up to four measurements around the apical half of the crown in each photograph were used to calculate an average enamel thickness; for the large specimen two photographs were used.

We could directly measure VEIW in all but two-sampled teeth (first Pmx and 11th Mx teeth in the small *Alligator*); these teeth were not considered further. We reconstructed tooth formation time and replacement rate from mean VEIW taken from direct measurements of the remaining samples.

### Transect orientation

**Figure 3:**
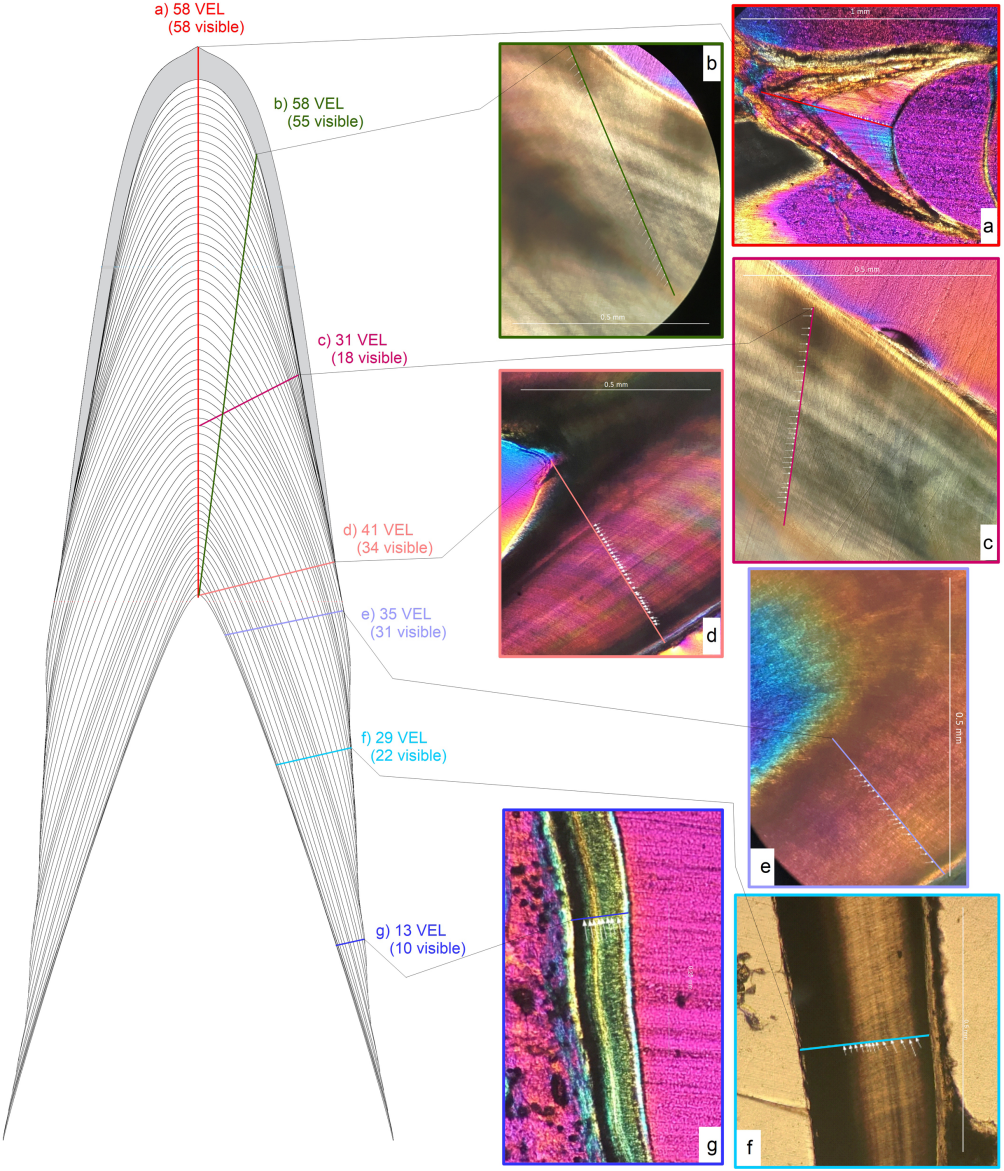
Schematic representation of transect orientations and examples. Schematic representation of different transect orientations. Transect orientations are represented by colored lines: (A) ****base-apex transect along the central axis (CA) from the tip of the pulp cavity to the crow apex representing CAH (red); (B) near-crown-apex transect measured at the tooth apex, yet not along the CA (yellow-green); (C) mid-crown transect measured in the central part of the crown (pink); (D) crown base transect from the tip of the pulp cavity to enamel dentin junction (EDJ) almost perpendicular to the CA (salmon); (E) root-base transect measured from the pulp cavity near the root base to the lateral dentin border (violet); (F) mid-root transect measured from the pulp cavity at the mid root to the lateral dentin border (sky blue); (G) root-apex transect measured from the pulp cavity near the root tip to the lateral dentin border (darker blue). Numbers next to the transects are the von Ebner lines crossed and the numbers visible under a hypothetical view through a microscope.

To determine the impact of sectioning strategy (intradental sampling effects) on calculation of von Ebner line counts, and specifically to test the assumption that mean VEIW is consistent regardless of the transect position used for sampling, we counted von Ebner lines across transects taken at multiple angles from the 12th Mx, 12th Dent, and 18th Dent tooth of the medium-sized *Alligator*. Transect positions were categorized as base-apex (Fig. 3A; from the tip of the pulp cavity to the crow apex (this is equal to the full CAH)); near-crown-apex (Fig. 3B; measured at the tooth apex, yet not along the central axis); mid-crown (Fig. 3C; measured in the central part of the crown); crown base (Fig. 3D; transect from the tip of the pulp cavity to enamel dentin junction almost perpendicular to the central axis); root-base (Fig. 3E; measured from the pulp cavity near the root base to the lateral dentin border); mid-root (Fig. 3F; measured from the pulp cavity at the mid root to the lateral dentin border); root-apex (Fig. 3G; measured from the pulp cavity near the root tip to the lateral dentin border). Although all of our transects were derived from longitudinal sections, transverse transects in longitudinal section are effectively equivalent to taking a transverse section itself, as long as the transverse section bisects the central axis (semi-)perpendicularly.

### Hypotheses testing

1. We tested the hypothesis that sampling transects taken in different orientations to von Ebner lines will not produce significantly different calculations of formation time by comparing VEIWs from transects taken perpendicular and oblique to von Ebner lines. We present the difference in formation time in days and in percent differences, and performed a paired *t*-test to test for significance.
2. We tested the hypothesis that measuring VEIWs at different distances from the pulp cavity will not result in significantly different mean VEIWs by comparing mean VEIW and resulting formation time calculations derived from eight distinct subsamples along the same transect in Dent 11 of the large specimen and mean VEIWs of three different sections along this transect. Subsamples were taken along the central axis at different distances from the pulp cavity (one apical, one basal and six in various parts of the mid crown region), using all recognizable VEIWs in each subsample. This approach attempts to capture variation in growth rate during the formation of a tooth using a central axis transect only. An ANOVA was performed to test the significance of the differences between the means of apical, basal and mid crown VEIWs and followed by a post-hoc Tukey–Kramer test comparing the VEIWs of the apical vs. mid crown, apical vs. basal, and mid crown vs. basal sections.
3. We tested the hypothesis that sampling transects taken in different regions of the tooth (with regard to the tooth from root to apex) will not produce significantly different VEIWs or different formation times (TFT) using multiple approaches. We assessed the impact of calculating formation time from transects located at regions around the tooth other than the central axis by calculating alternative formation times using a mean VEIW and transect length from a region of the tooth off the central axis and dividing this by a formation time derived from a central axis transect (= true formation time, where both mean VEIW and CAH were derived along the central axis). A mean of these values was used to determine what percentage of the true formation time was captured by formation times taken from transects not on the central axis.

$$\eqalign{ & {\rm TFT\,(a)}={\rm transect\,(a)}/{\rm mean\,VEIW}\,({\rm region\,a}), {\rm a}\rightarrow {\rm n}; \cr & {\rm TFT\,(true)}= {\rm CAH}/{\rm mean\,VEIW}({\rm CAH}) \cr & {\rm TFT}\,({\rm a} \rightarrow {\rm n})/{\rm TFT}({\rm true})={\rm Y}; {\rm Y}\times 100 = [\%] \cr }$$

We also assessed the impact of subsampling along transects not located along the central axis. For this, we calculated mean VEIW from subsamples of three transects closest and most distant from the central axis and calculated formation times using CAH derived from a central axis transect. These mean VEIWs represent extreme end members for VEIW thickness primarily related to tooth geometry. We express the difference in the formation time estimates as a percent calculated by dividing the formation time based on the portion furthest from the central axis by the formation time obtained from the portion closest to the central axis. We test the significance of the difference between the VEIWs from close and most distant to the central axis with a two-sample *t*-test assuming unequal variances.

$$\eqalign{ & {\rm{TFT}}({\text{near CA}}) = {\rm{CAH}}/{\rm{mean VEIW}}({\text{near CA}}); \cr & {\rm{TFT}}({\text{far CA}}) = {\rm{CAH}}/{\text{mean VEIW}}({\text{far CA}}) \cr & {\rm{TFT}}({\text{far CA}})/{\rm{TFT}}({\text{near CA}}) = {\rm{Y}};{\rm{Y}} \times 100 = [{\rm{\% }}] \cr} $$

Additionally, the change in resolution of von Ebner line with increasing distance from the pulp cavity in lateral transects was tested in a theoretical framework by creating a figure (Fig. 3) with von Ebner lines in the same orientations and relative distances from each other and counting the number of von Ebner line crossed by transects drawn in the tooth and the number of von Ebner line visible under a magnification that mimics the resolution achieved under a microscope.

4. We tested the hypothesis that sampling VEIW at different tooth positions within the jaw will not produce different calculations of formation time in two ways. First, the mean VEIWs for all sampled tooth positions obtained from transects with a similar orientation were subjected to an ANOVA to test for significant differences between their means. To test the amount of error a researcher could encounter if using the mean VEIW derived randomly from anywhere in the dentition to calculate the formation times of the remainder of the dentition, we calculated the grand mean standard deviation of the VEIW derived from all transects taken along the central axis and applied this SD range to the VEIWs used to calculate formation times. The grand mean standard deviation is subtracted from the mean VEIW of the tooth to obtain a minimum VEIW and added to obtain a maximum VEIW. Tooth formation time is derived from dividing the CAH (from the tip of the pulp cavity to crown apex) by those VEIWs. The fit of the minimum and maximum mean VEIW based on formation times was compared to the true formation times of the sampled teeth by performing a two-sample *t*-test for unequal variances in MS Excel (Excel for Office 365 16.0.11328.20362). The difference between the true formation time and the minimum and maximum formation times are shown in days and percent differences.
5. We tested the hypothesis that sampling during different growth stages of an organism’s life history will result in different calculations of mean VEIW by investigating the relationship between VEIW and body length and tooth replacement rate and body length using reduced major axis regression, utilizing the RSQ function to derive *R*^2^ and the FDIST function to arrive at a significance *p*. For other plots the coefficient of determination (*R*^2^) was determined using the inbuilt functionality of MS Excel (Excel for Office 365 16.0.11328.20362) for charts. Additionally, we performed an ANOVA on the same dataset created for testing hypothesis 4, but here grouped by ontogenetic stage. Using the specimens as groups we can make inferences about the presence or absence of differences in the mean VEIW during ontogeny.

### Application to fossil taxa

We used the mean standard deviation of *Alligator* to derive an estimated SD for the tooth formation times calculated for archosaurs in Erickson (1996b) and Garcia & Zurriaguz (2016) both of which reported VEIW ranges, but no SDs. We also used the reported SD for several theropods in D’Emic et al. (2019) as an opportunity to test the efficacy of applying the relative standard deviation of *Alligator* to other archosaurs when not enough data on VEIW is available to confidently derive SD on the raw data. For this we compared actual SD values calculated in D’Emic (control values) for the theropods *Majungasaurus*, *Ceratosaurus*, and *Allosaurus*, to SD estimates for those same taxa that we derived by applying the relative standard deviation from *Alligator* (approximated values). CAH (from the tip of the pulp cavity to the crown apex) was derived by multiplying the reported mean VEIW with the reported tooth formation time. The CAH was then divided by the VEIW plus or minus SD to derive upper and lower ends to the range of possible tooth ages.

We also explore the effect of applying the relative standard deviation of *Alligator* on calculations of tooth formation time and tooth replacement rate in sauropods using mean VEIW ± 1SD. We followed a similar approach using data from D’Emic et al. (2013), D’Emic & Carrano (2019), Sattler (2014), Kosch (2014), Schwarz et al. (2015). For the sauropod data, we calculated the resulting minimum, maximum, and true formation times and replacement rates, and summarized these data for each tooth position (functional, replacement tooth one, replacement tooth two, etc.). This representation of tooth formation time and replacement rate is analogous to how these values are calculated in D’Emic et al. (2013, 2019), Sattler (2014), Kosch (2014), and Schwarz et al. (2015). All originally reported formation times in these publications are based on the transfer functions of tooth height to formation time of D’Emic et al. (2013), using the function for narrow crowned taxa derived from sampling *Diplodocus* premaxillary teeth for *Dicraeosaurus* and *Tornieria* and the function for broad crowned taxa derived from *Camarasaurus* premaxillary teeth for *Giraffatitan* and *Brachiosaurus*. For all teeth, a mean VEIW of 15 µm was assumed, derived from D’Emic et al. (2013) calculation of mean VEIW for both *Dipldocus* and *Camarasaurus*.

As reported, tooth replacement rates represent a mean value derived from all replacement rates that could be obtained (and might differ slightly from tooth replacement rates that are calculated subtracting the mean tooth formation times of those positions from one another due to empty tooth positions in some tooth families). Tooth replacement rate max-max is derived using VEIW − 1SD to calculate maximal formation time for both teeth in a tooth family. Replacement rate min-min is derived using VEIW + 1SD to calculate minimal formation time for both teeth in a tooth family. Replacement rate min-max and max-min are derived by subtracting formation times calculated using VEIW + 1SD and VEIW − 1SD. Tooth replacement rate min-max applies VEIW + 1SD to calculate minimum tooth formation time for the oldest tooth in a successional pair of teeth in a tooth family and VEIW − 1SD for the youngest tooth in the pair, whereas tooth replacement rate max-min, takes the opposite approach. We obtained the mean tooth formation time and mean values from tooth replacement rates for each tooth position for the completely sampled dentitions of *Tornieria*, *Giraffatitan* and *Dicraeosaurus*. Whereas for the more sporadically sampled dentitions of *Camarasaurus* (UMNH 5527) and *Diplodocus* (YPM 4677) and *Brachiosaurus* (USNM 5730) we present the range of reported formation times for each tooth position in the Pmx or Mx and Dent.

## Results

### Intradental effects

#### Sampling transects taken in different orientations to von Ebner lines (perpendicular or obliquely oriented to von Ebner lines) (Figs. 2, 3 and 4) will produce significantly different calculations of tooth formation time

**Figure 4:**
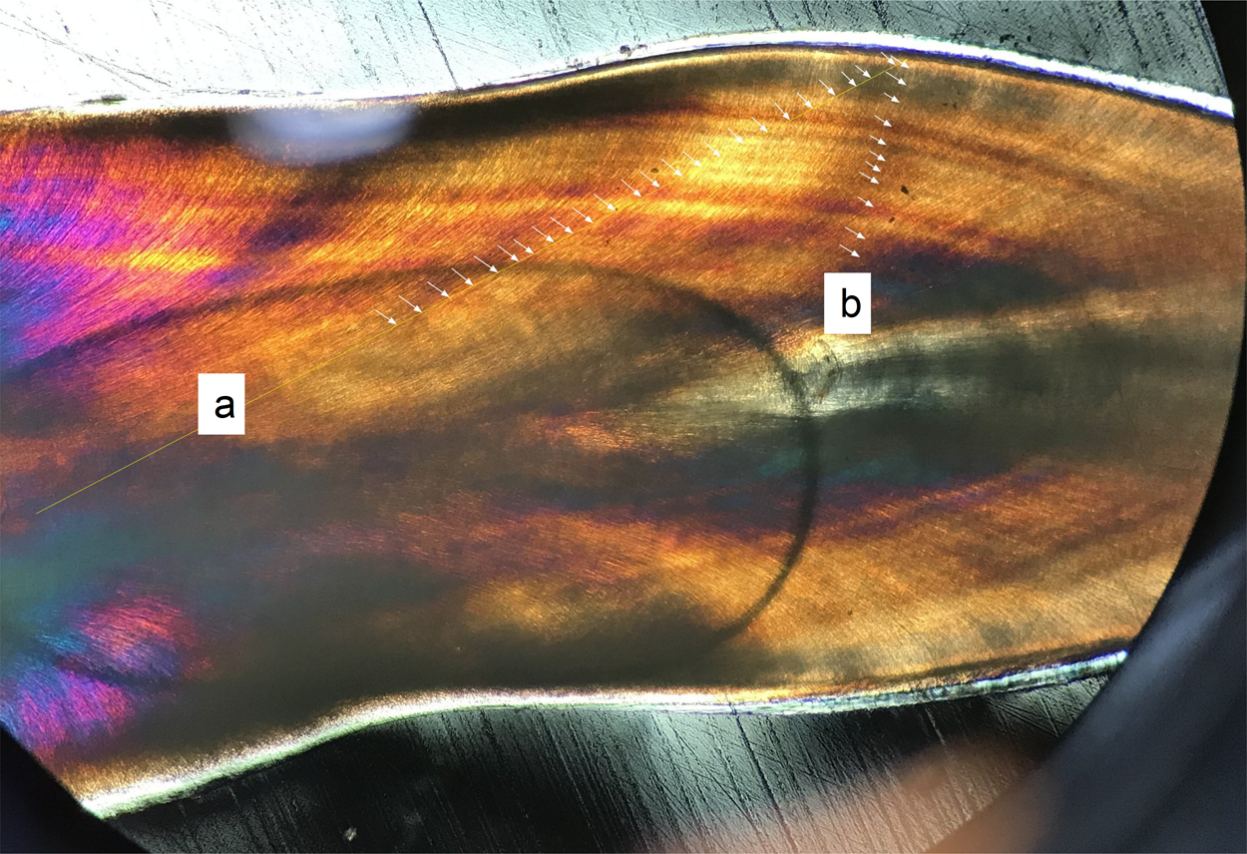
Oblique transect orientation. Yellow lines mark transects. Individual von Ebner lines (VEL) are marked with arrows where they intersect with the transect: (A) Transect strongly oblique to VEL, reaching from the crown base near the pulp cavity to the mid-crown (with no visible VEL in the medial position of the tooth); (B) transect perpendicular to VEL. Both transects derive from the first premaxillary alveolus of the medium sized *Alligator* (NCSM 100804).

Our data rejects the hypothesis that measuring oblique to von Ebner line orientation will not significantly affect calculations of tooth formation time. Transects that cross von Ebner lines obliquely (Figs. 3 and 4; File S1) (e.g., top of the pulp cavity to the enamel dentin junction, in areas adjacent to the apex (Fig. 3B)) yield VEIWs up to twice the width as those made perpendicular to von Ebner line trendlines. Table 1 illustrates these differences with 33–20% shorter tooth formation times calculated when mean VEIW is derived from obliquely oriented measurements compared to those based on perpendicularly oriented measurements when crown height is derived from the CAH for three pairs of oblique and perpendicular series of measurements. This is a significant difference, paired *t*-test, *t*_2_ = 10.36 (*t*_crit_ = 4.30), *p* = 0.009, mean difference 64 days. However, if a researcher were to use the *same* transect to derive mean VEIW and transect length in calculations of tooth formation time the effect is less. Oblique transects result in both a larger mean VEIW and a longer transect, whereas perpendicular transects are composed of shorter VEIWs across a shorter transect. Nevertheless, both approaches underestimate age compared to transects along the true central axis because they fail to account for the apical-most component in derivations of CAH (Fig. 2) (see hypothesis 3 below).

**Table 1:** Transects oblique and perpendicular to von Ebner lines. Three pairs of measurements, each from the same tooth with one oblique and one perpendicular to the von Ebner lines and the resulting TFT for the tooth from applying them to central axis height.

Tooth	Oblique					Perpendicular					
	Mean VEIW (µm)	SD	Min	Max	TFT (days)	Mean VEIW (µm)	SD	Min	Max	TFT (days)	TFT difference
Medium (NCSM 100804) Dent 18	21	5	12	34	118.45	14	4	8	21	177.68	59.22
Large (NCSM 100805) Dent 11	21	6	12	29	239.29	17.5	3	13	23.5	295.59	56.30
Large (NCSM 100805) Dent 11	22	6	12	36	228.41	16.5	3	12.5	21.5	304.55	76.14

**Note:**

SD, standard deviation; TFT, tooth formation time; VEIW, von Ebner line increment width.

#### Measuring VEIWs at different distances from the pulp cavity along the central axis will not result in significantly different calculations of mean VEIW

**Figure 5:**
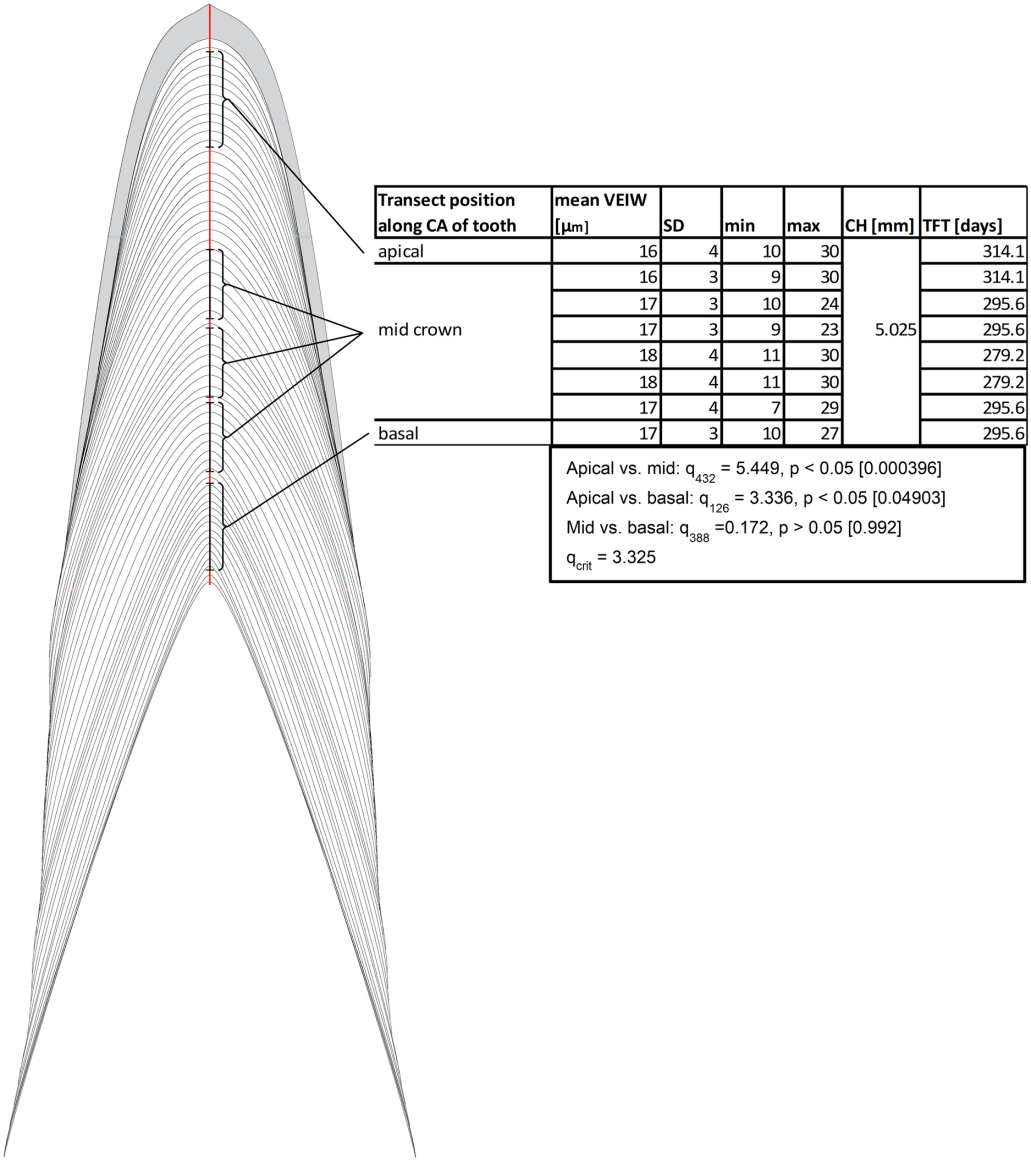
Examples of transects in different distances to pulp cavity. Schematic representation of transects in different distance to the pulp cavity. All transects are along the Central axis from the tip of the pulp cavity to the crown apex. All examples are from Dent 11 of the large specimen (NCSM 100805). Abbreviations: CA, central axis; CH, crown height; TFT, tooth formation time; VEIW, von Ebner line increment width.

Our data support the hypothesis that there is no consistent, statistically significant difference between calculations of tooth formation times derived from measuring VEIWs at different distances from the pulp cavity along the central axis. Calculations only vary by 11–12% (Fig. 5) and we find conflicting statistical results depending on approach and methods. In the case of the eight transects presented in Fig. 5 an ANOVA between the 86 VEIWs measured for the crown apex, the 348 VEIWs of the combined six mid crown transects, and the 42 VEIWs of the crown base finds significant differences between the VEIWs in the three regions of the tooth, *F*_2, 473_ = 7.52 (*F*_crit_ = 2.62), *p* = 0.0006. However, the post-hoc Tukey–Kramer test of the VEIWs of the regions only finds significant difference between the VEIWs of the apical transect and the six mid-crown transects and between the apical VEIWs vs. basal VEIWs. We find no significant difference between mid crown VEIWs and basal VEIWs (Fig. 5). Sampling transects taken in different regions of the tooth (with regard to the tooth from rop to apex) will produce significantly different VEIWs.

We find transect position critical for precise estimates of mean VEIW and tooth formation time and therefore our data refutes the hypothesis that sampling transects taken in different regions of the tooth (with regard to the tooth from root tip to apex) will not produce significantly different VEIWs. These data contradict the assumption that mean VEIW is consistent regardless of the transect position used for sampling. With regard to location on the tooth, we find that sections that do not precisely section the central axis do not preserve all von Ebner lines. Transverse sections omit von Ebner lines, particularly those of the crown apex (Fig. 2). This combined with the lack of resolution of von Ebner line further from the pulp cavity results in much shorter calculated tooth formation times (tooth formation times calculated with this approach are only 83% on average of those derived from a central axis transect in the medium *Alligator*). We also find that mean VEIW in a tooth decreases laterally (Figs. 2 and 3; File S1) when sampled perpendicular to von Ebner line trendlines; VEIWs are thickest along the central axis from above the pulp cavity to the crown apex and thinnest near the enamel dentin junction in a transversal plane. The differences between these two groups are significant, *t*_80_ = 5.40 (*t*_crit_ = 1.99), *p* = 6.61451E−07. Table 2 exemplifies this difference in VEIW thickness and resulting estimates if tooth formation time is derived from VEIWs obtained from subsections of lateral transects other than the central axis are then applied to CAH. VEIW can be incorrectly measured by up to 36%, which would lead to inaccurately calculated tooth formation time and replacement rates.

**Table 2:** VEIW decrease in marginal von Ebner lines. Three examples of lateral transects other than the CA. Each has a subsample with a number of VELs close to the CA with their mean VEIW and a subsample of VEL furthest from the CA with their mean VEIW.

Tooth	Transect	Mean VEIW near CA (µm)	Based on # VEL	TFT based on near CA VEIW	Mean VEIW far CA (µm)	Based on # VEL	TFT based on far CA VEIW	Difference in TFT estimates (%)
Medium (NCSM 100804) Dent 18	mid crown	17.5	10	142.14	13.5	15	184.26	77.14
	mid crown	18.4	10	135.19	13.9	19	178.35	75.80
Large (NCSM 100805) Dent 4	root base	19.3	26	666.30	12.1	9	1,062.25	62.73

**Note:**

CA, central axis; TFT, tooth formation time; VEIW, von Ebner line increment width; VEL, von Ebner line.

### Intramandibular effects

#### Sampling different tooth positions within the jaw will not produce significantly different calculations of tooth formation time

**Figure 6:**
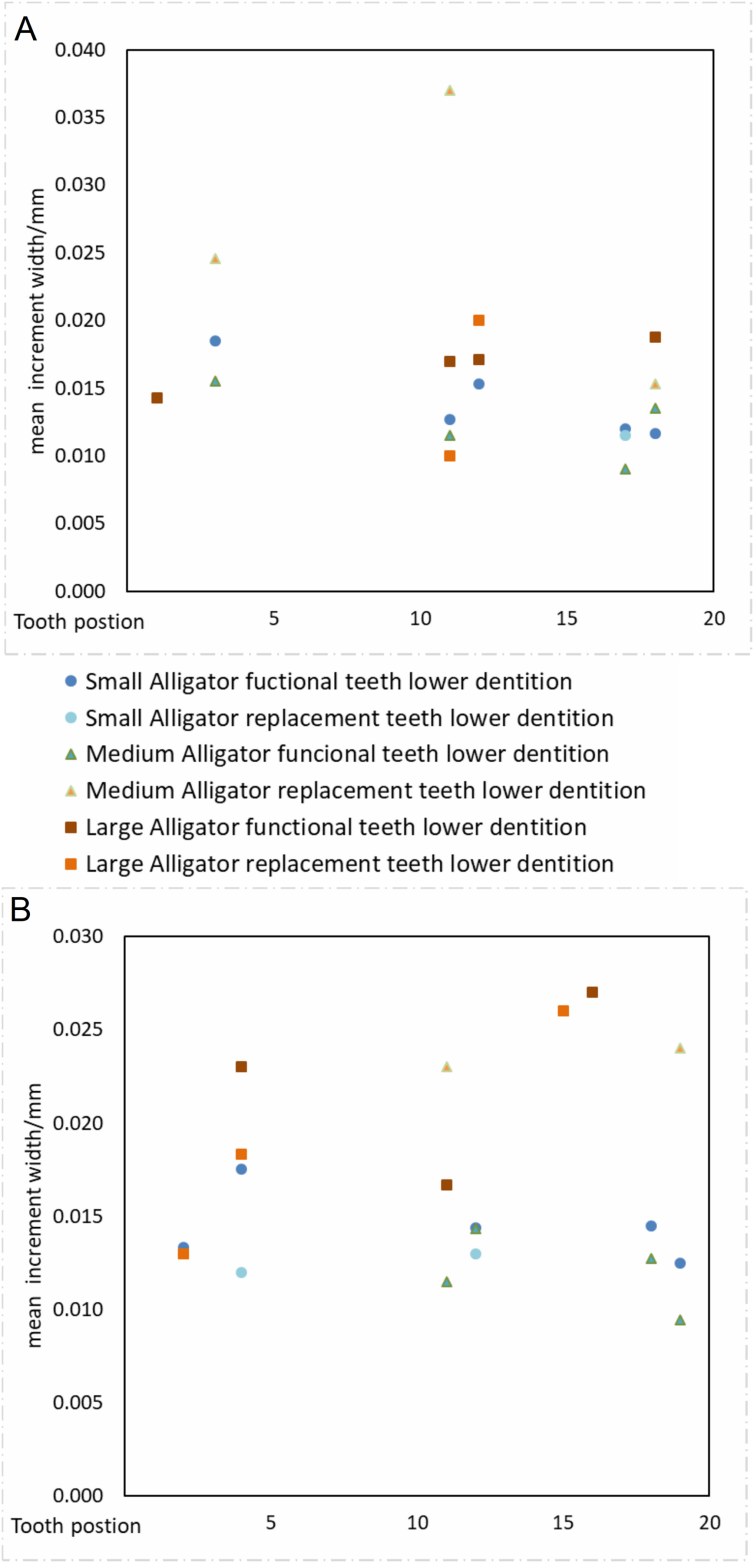
VEIW according to tooth position. Tooth position on the *x* axis, VEIW width in mm on the *y* axis. Each tooth position is depicted by its own point. (A) Dentition of the upper jaw. (B) Dentition of the lower jaw.

Our data supports the hypothesis that sampling different tooth positions within the jaw will not produce significantly different calculations of tooth formation time. Despite considerable variation in our calculations of VEIW values (ranging 5–39 µm within the sample and 6–38 µm within a single tooth), we find no statistical difference between mean VEIWs of tooth positions calculated across the tooth row (Table 3; Fig. 6), one-way ANOVA, *F*_10, 19_ = 1.04 (*F*_crit_ = 2.38), *p* = 0.448. If only the alveoli with three or more samples for an alveolus position (4 in alveolus 4, 6 in alveolus 11, 5 in alveolus 5, 4 in alveolus 18) are taken into account there is even less of a difference recovered, one-way ANOVA, *F*_3, 15_ = 0.48 (*F*_crit_ = 3.29), *p* = 0.698. The lack of systematic variation of VEIW in teeth of different mandibular quadrants or between teeth of the lower and upper tooth rows, supports the assumption that data derived from one tooth position can be applied to a tooth from a different tooth position with reliability.

**Table 3:** VEIW according to tooth position.

	Tooth position	1	2	3	4	11	12	15	16	17	18	19
Small Alligator NCSM 100803	Functional teeth upper dentition			0.019		0.0127	0.0153			0.0120	0.0117	
	Replacement teeth upper dentition									0.0115		
	Functional teeth lower dentition		0.0133		0.0175		0.0144				0.0145	0.0125
	replacement teeth lower dentition				0.0120		0.0130					
Medium Alligator NCSM 100804	Functional teeth upper dentition			0.0156*		0.0115*				0.009′	0.0135′	0.0094*
	Replacement teeth upper dentition			0.0246		0.0370					0.0153	0.0240
	Functional teeth lower dentition					0.0115*	0.0143*				0.0128*	
	Replacement teeth lower dentition					0.0230						
Large Alligator NCSM 100805	Functional teeth upper dentition	0.0143			0.0230	0.0170	0.0171				0.0188	
	Replacement teeth upper dentition		0.0130		0.0183	0.0100	0.0200					
	Functional teeth lower dentition					0.0167			0.0270			
	Replacement teeth upper dentition							0.0260				

**Note:**

Mean VEIW for the sampled tooth positions in mm. Only measurements with the same transect orientation were used (central axis, excerpt for ′ root base; * a combination of root base and crown base transects). Some tooth positions were sampled but had a different transect orientation from the other teeth and were excluded from table and analyses. Second generation replacement teeth (one in the dentition of the smallest specimen, four in the dentition of the medium sized specimen, three in the dentition of the largest specimen) are excluded.

**Figure 7:**
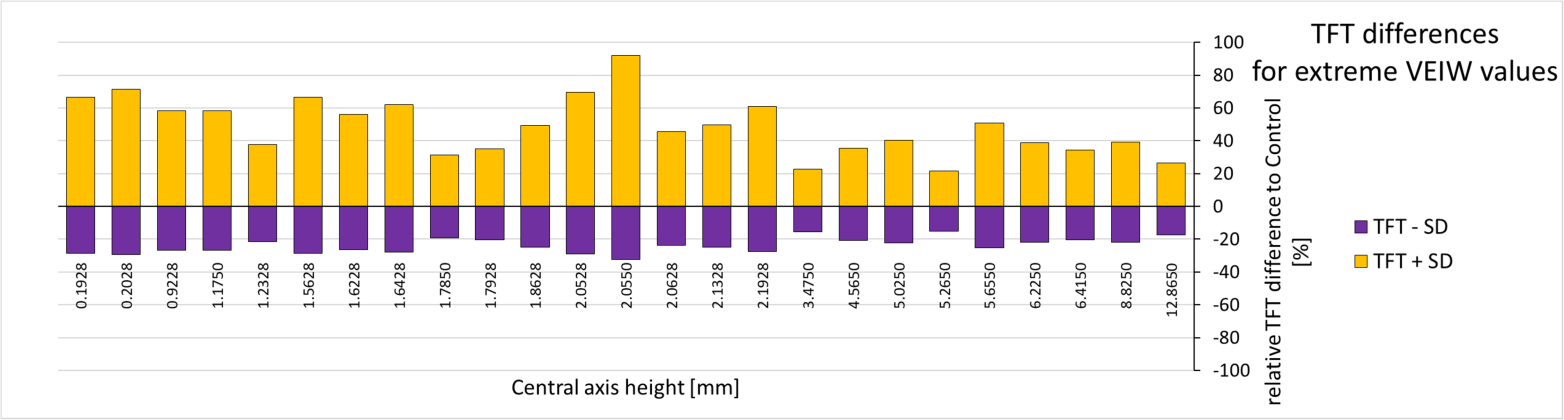
Tooth formation time differences for extreme VEIW values. For each tooth with transects along the central axis (on the *x*-axis with their central axis height) from all our specimens the deviation of the upper and lower TFT estimate (upper estimate: based on mean VEIW (tooth) − 1SD (grand mean); lower estimate based on mean VEIW (tooth) + 1SD (grand mean)) to the calculated TFTs (based on mean VEIW (tooth)) is shown in percent (see last two rows of Table 4). Lower TFT estimates are in purple, upper TFT estimates in yellow. Abbreviations: SD, standard deviation; TFT, tooth formation time; VEIW, von Ebner line increment width.

As would be expected, the smaller the mean VEIW and the longer the CAH of the teeth under study, the more pronounced the influence the static grand mean standard deviation has on derived tooth formation times (Table 4; Fig. 7). Thus we find it is the size of the tooth that makes the tooth formation time differences more or less pronounced on an absolute scale, not the position within the jaw. The (grand mean) SD of all VEIW measurements along the central axis is 0.00479 mm, which is 29.94% of the mean VEIW of 0.0160 mm. On average tooth formation times calculated from mean VEIW − 1SD are 48.78% bigger whereas those calculated from mean VEIW + 1SD are 23.94% smaller. When tooth specific SDs are used these values change to 41.23% and 20.62% respectively (see Table S1). Two-sample, one sided *t*-tests between the unmodified tooth formation times and tooth formation time calculated from mean VEIW ± 1SD assuming unequal variances find a significant difference between tooth formation time calculated from mean VEIW − 1SD and the unmodified tooth formation time (*p* = 0.041) but no significant difference for tooth formation time calculated from mean VEIW + 1SD (Table 4). Whereas, no significant differences were found when using the tooth specific SDs instead of a grand mean standard deviation (Table S1).

**Table 4:** Tooth formation times based on mean VEIW (tooth) ± 1SD (grand mean).

Crown height (mm)	0.193	0.203	0.923	1.175	1.233	1.563	1.623	1.643	1.785	1.793	1.863	2.053	2.055	2.063	2.133
TFT by sampled CA sections (days)	16.067	17.635	70.985	90.385	70.446	130.233	121.710	131.424	89.250	96.908	128.469	175.954	205.500	134.530	148.111
VEIW of the tooth (mm)	0.012	0.012	0.013	0.013	0.018	0.012	0.013	0.013	0.020	0.019	0.015	0.012	0.010	0.015	0.014
TFT + SD (days)	26.739	30.222	112.395	143.112	96.992	216.745	189.942	213.065	117.354	130.763	191.837	298.502	394.409	195.644	221.928
TFT − SD (days)	11.483	12.450	51.873	66.050	55.308	93.081	89.544	95.016	72.006	76.978	96.570	124.742	138.948	102.510	111.143
SD + (days)	10.673	12.587	41.410	52.728	26.546	86.511	68.232	81.641	28.104	33.855	63.368	122.548	188.909	61.113	73.817
SD − (days)	−4.583	−5.185	−19.112	−24.335	−15.138	−37.152	−32.166	−36.408	−17.244	−19.930	−31.899	−51.212	−66.552	−32.021	−36.968
SD + (%)	66.428	71.378	58.337	58.337	37.683	66.428	56.061	62.120	31.490	34.935	49.326	69.648	91.926	45.427	49.839
SD − (%)	−28.528	−29.403	−26.924	−26.924	−21.488	−28.528	−26.429	−27.703	−19.321	−20.566	−24.830	−29.105	−32.385	−23.802	−24.960

Crown height (mm)	2.193	3.475	4.565	5.025	5.265	5.655	6.225	6.415	8.825	12.865	Mean	Variance	*t*-statistic	*p*-value
TFT by sampled CA sections (days)	173.116	133.654	249.000	301.500	195.000	396.842	364.035	342.133	519.118	559.348	194.454	20739.047		
VEIW of the tooth (mm)	0.013	0.026	0.018	0.017	0.027	0.014	0.017	0.019	0.017	0.023				
TFT + SD (days)	278.380	163.835	337.058	423.087	237.052	597.759	505.673	459.516	722.749	706.467	280.449	37706.375	1.7786	0.0411
TFT − SD (days)	125.616	112.863	197.423	234.197	165.620	297.012	284.381	272.519	405.009	462.942	150.211	13402.557	−1.1972	0.1187
SD + (days)	105.264	30.181	88.058	121.587	42.052	200.917	141.638	117.383	203.631	147.119				
SD − (days)	−47.500	−20.791	−51.578	−67.303	−29.380	−99.831	−79.654	−69.615	0.000	−96.406				
SD + (%)	60.806	22.582	35.365	40.327	21.565	50.629	38.908	34.309	39.226	26.302				
SD − (%)	−27.438	−15.556	−20.714	−22.323	−15.067	−25.156	−21.881	−20.347	−21.981	−17.235				

**Note:**

TFTs based on mean VEIWs for the sampled tooth positions sorted by ascending CAH. Rows TFT ± SD show the TFT based on VEIW ± SD (grand mean) derived for each tooth. TFT + SD shows the mean VEIW (tooth) − 1SD (grand mean) (TFT + SD, as it produces bigger TFTs); TFT − SD shows VEIW (tooth) + 1SD (grand mean) (TFT − SD as it produces smaller TFTs). SD ± (days) show the differences of TFT ± SD to the baseline TFT. Rows SD ± (%) are showing the same differences in percent (see also Fig. 7). The last four columns show the mean TFT, the variance, *t*-test statistic and the *p*-value for a one sided two-sample *t*-test comparing TFT + SD and TFT − SD. SD, standard deviation; TFT, tooth formation time; VEIW, von Ebner line increment width.

#### Ontogenetic effects: Sampling during different growth stages of an organism’s life history will not result in different calculations of mean VEIW

**Figure 8:**
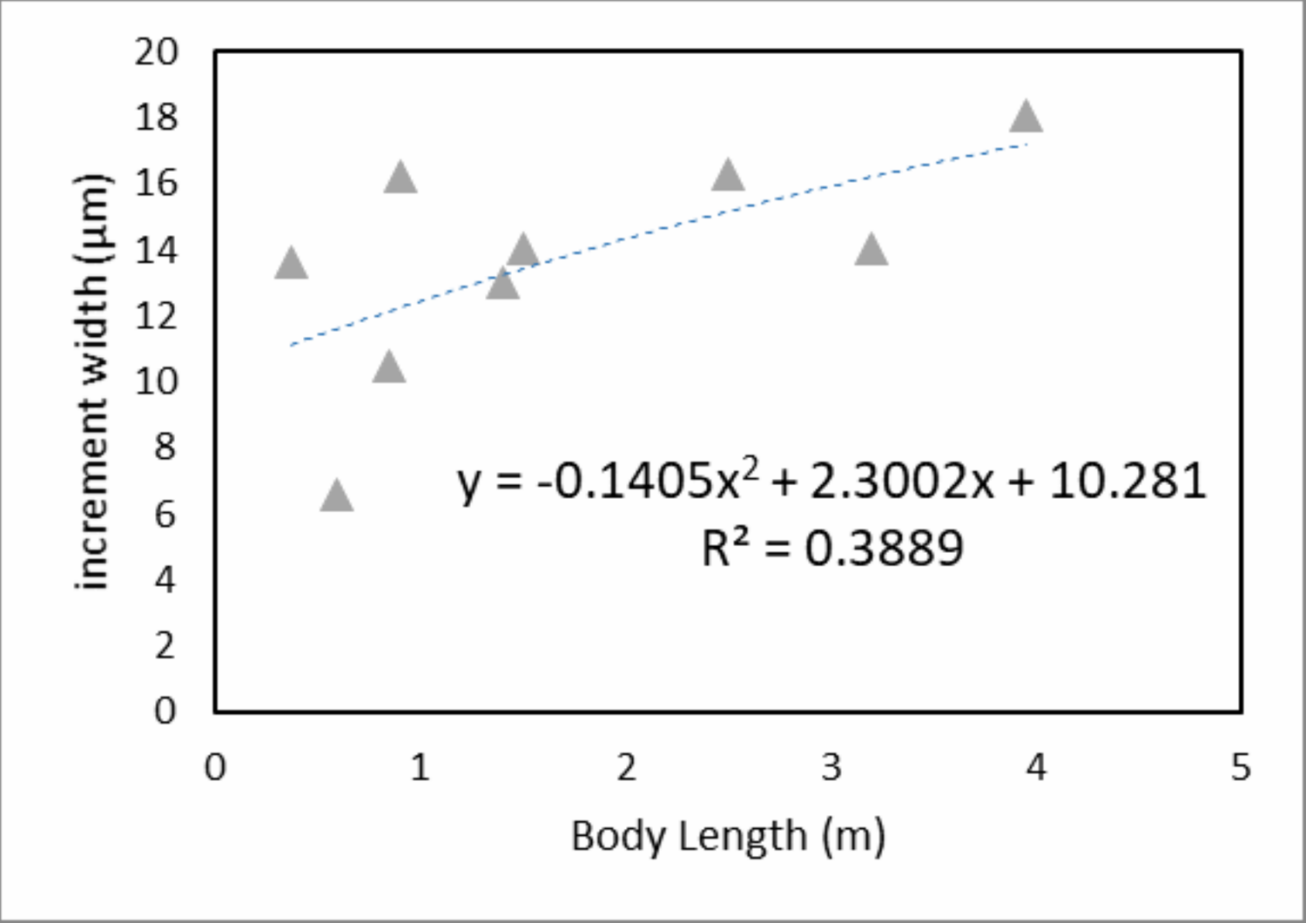
Mean specimen VEIW by body length. Mean VEIW vs. body length for *Alligator* specimens from this study and Erickson (1992, 1996a). The nearly linear shaped quadratic regression and the coefficient of determination are displayed.

When we derive mean VEIW for individual teeth with the same transect orientation and location and compare them among the sampled individuals (Fig. 6; Table 3), we do not observe significant differences between individuals of different ages and body sizes in our sample (one-way ANOVA, *F*_2, 35_ = 2.29 (*F*_crit_ = 3.27), *p* = 0.116). This supports the hypothesis that sampling during different growth stages will not result in significantly different calculations of mean VEIW. When we derive mean VEIW across the whole dentition of just our sampled individuals (from smallest to largest: NCSM 100803, NCSM 100804, and NCSM 100805), we find a slight increase during ontogeny (Fig. 5A in File S1); however, three individuals are not enough for a meaningful statistical comparison. When we add the mean VEIW of differently sized *Alligator* specimens ranging in body length from 0.60 m to 3.20 m derived from Erickson (1992, 1996a), we find the relationship between VEIW and body length shows a weak ontogenetic signal (*R*^2^ = 0.389) that still does not reach the level of 95% significance (*p* = 0.074) in an reduced major axis regression (Fig. 5B in File S1; Fig. 8).

### Application to fossil taxa

Table 5 gives the error margins for tooth formation times of various archosaurs included in the studies of Erickson (1996b), Garcia & Zurriaguz (2016), and D’Emic et al. (2019). Garcia & Zurriaguz (2016) only reported highest and lowest mean VEIWs and highest and lowest tooth formation times from their five-tooth sample, but never made clear if those values are directly related, pairing the lowest mean VEIW with the highest tooth formation time, and they provided no measurements for the five teeth sampled. We assume in Table 5 that the highest age corresponds to the smallest VEIW and vice versa. The VEIW in these publications likely underestimate the VEIW as it would be measured along the central axis, because Erickson (1996b), Garcia & Zurriaguz (2016), and in part D’Emic et al. (2019) based their calculations of VEIW on a combination of transverse sections and/or multiple measurements along transects perpendicular or semi-perpendicular to the central axis and the von Ebner lines (see Fig. S1; Fig. 1B). We did not include the fossil crocodylians with reported VEIWs and tooth formation times from Ricart et al. (2019) because they based their tooth formation time calculations on the radius of dentine in their transverse sections instead of the CAH, which means they are not representative of actual tooth formation times, whereas the tooth formation times reported by Erickson are supported by von Ebner line counts for all regions where they were possible.

**Table 5:** Error margins for published Tooth formation times.

Taxon	Mean increment width (µm)	Central axis height (from mean VEIW and TFT) (mm)	Mean tooth formation time (days)	VEIW SD based on *Alligator* (µm)	TFT + SD (days)	TFT − SD (days)	SD + (days)	SD − (days)	Source
Crocodilian	13	3.1980	246	3.8916	351.1046	189.3248	105.1046	−56.6752	Erickson (1996b)
*Leidyosuchus*	19	5.3770	283	5.6877	403.9131	217.8005	120.9131	−65.1995	
Adult *Edmontonia*	13.5	3.7665	279	4.0413	398.2040	214.7221	119.2040	−64.2779	
Adult *Triceratops*	15.8	6.0198	381	4.7298	543.7840	293.2226	162.7840	−87.7774	
Infant Lambeosaurinae	11	1.6170	147	3.2929	209.8064	113.1331	62.8064	−33.8669	
Adult *Prosaurolophus*	16	5.1680	323	4.7897	461.0032	248.5850	138.0032	−74.4150	
Infant *Maiasaura*	12	1.5840	132	3.5923	188.3976	101.5889	56.3976	−30.4111	
Adult *Maiasaura*	13	3.6530	281	3.8916	401.0585	216.2613	120.0585	−64.7387	
Juvenile *Edmontosaurus*	14	3.1500	225	4.1910	321.1323	173.1629	96.1323	−51.8371	
Adult *Edmontosaurus*	19.8	6.7122	339	5.9272	483.8393	260.8988	144.8393	−78.1012	
Adult *Deinonychus*	10.1	4.1713	413	3.0235	589.4562	317.8502	176.4562	−95.1498	
Adult *Troodon*	11	3.9930	363	3.2929	518.0934	279.3696	155.0934	−83.6304	
Juvenile Albertosaur	14.5	4.9155	339	4.3406	483.8393	260.8988	144.8393	−78.1012	
Adult Albertosaur	14	7.2660	519	4.1910	740.7451	399.4292	221.7451	−119.5708	
Juvenile *Tyrannosaurus*	14	3.6960	264	4.1910	376.7952	203.1779	112.7952	−60.8221	
Sub. Ad. *Tyrannosaurus*	14	4.3960	314	4.1910	448.1579	241.6585	134.1579	−72.3415	
Adult *Tyrannosaurus*	17	15.8610	933	5.0890	1331.6286	718.0490	398.6286	−214.9510	
Titaosauria indet.	18.2	1.1648	64	5.4482	91.3443	49.2552	27.3443	−14.7448	Garcia & Zurriaguz (2016)
MPCA-Ph 4, 5, 9, 13, 19	20.8	1.1232	54	6.2266	77.0717	41.5591	23.0717	−12.4409	
*Majungasaurus*									D’Emic et al. (2019)
FMNH PR 2100 Pmx dext 3	17.55	5.1249	292	5.2540	416.7583	224.7270	124.7583	−67.2730	
UA 9944 Mx sin 1	17.55	4.6335	264	5.2540	376.7952	203.1779	112.7952	−60.8221	
FMNH PR 2100 Dent dext 2	17.55	4.3878	250	5.2540	356.8137	192.4033	106.8137	−57.5967	
*Ceratosaurus*									
BYU 12893 Mx dext 6	14.46	4.8312	334	4.3300	476.7030	257.0508	142.7030	−76.95	
*Allosaurus*									
BYU 8901 Pmx dext 2	23	8.0500	350	6.8851	499.5391	269.3646	149.5391	−80.6354	
BYU 8901 Mx dext 6	23	7.3600	320	6.8851	456.7215	246.2762	136.7215	−73.7238	
BYU 2028 Dent sin 5	23	8.1650	355	6.8851	506.6754	273.2127	151.6754	−81.7873	

**Note:**

Published TFTs from Erickson (1996b), Garcia & Zurriaguz (2016), and D’Emic et al. (2019) ± one standard deviation (SD) based on relative SD. Central axis height was not included in the original publications and was back calculated based on mean VEIWs given in the publications. The last two columns are showing just the differences of TFT from VEIW ± SD to mean TFT. SD, standard deviation; TFT, tooth formation time; VEIW, von Ebner line increment width.

As is to be expected, the greater the CAH, the greater the variation in tooth formation time produced by using mean VEIW ± 1SD. In an adult *Tyrannosaurus* with more than 15 mm CAH (from the tip of the pulp cavity to the crown apex) the difference between minimum and maximum estimates of tooth formation time is greater than a year and a half (613 days). Whereas, in the much shorter titanosaur tooth in our sample this difference is little more than 5 weeks (36 days). Table 6 summarizes the comparison of the SD values reported by D’Emic et al. (2019) and the SD values based on the SD range of *Alligator* we calculated.We also explore the effect of applying the relative standard deviation of *Alligator* on calculations of tooth formation time and replacement rate in sauropods (Table 7) using mean VEIW ± 1SD. Even with the range of tooth replacement rate min-min and max-max generated by using VEIW ± 1SD the replacement rate values for narrow crowned taxa (*Diplodocus*, *Torneria*, *Dicraeosaurus*) and broad crowned taxa (*Camarasaurus*, *Brachiosaurus*, *Giraffatitan*) barely overlap, with the exception of the younger generation teeth (r3 or younger) of *Dicraeosaurus*, which are calculated to have longer replacement rates than younger teeth of their tooth families. Tooth formation times based on VEIW ± 1SD between broad and narrow crowned taxa have more overlap. In all cases except for the distalmost tooth position in *Dicraeosaurus*, we derive a negative number for a mean tooth replacement rate using tooth replacement rate min-max (VEIW + 1SD to calculate minimum formation time for the oldest tooth in a successional pair of teeth in a tooth family and VEIW − 1SD for the youngest tooth in the pair), indicating that the “younger” replacement tooth (r2) would have formed before the “older” replacement tooth (r1), which is not biologically feasible. We caution against seeing this as a failure of using tooth formation times based on mean VEIWs to derive replacement rates. It is rather unlikely that all VEIWs in a tooth will be at the upper end of the VEIW range and VEIWs in the preceding tooth in the lower VEIW range. Such extremely divergent VEIWs in teeth of a tooth family are deemed highly unlikely, especially if both teeth are still growing replacement teeth subjected to the same environmental factors affecting the organism forming parts of both teeth at the same time.

**Table 6:** Error margin differences for theropod VEIWs.

Taxon	Mean VEIW (µm)	# transects	# measured VEIWs	Previously reported VEIW SD (µm)	*Alligator* based VEIW SD (µm)	Difference old–new VEIW SD (%)
*Alligator* average	16.00	87	4654	–	4.79	–
*Majungasaurus* average	17.55	54	544	5.15	5.25	2.04
*Ceratosaurus* average	14.46	9	54	3.26	4.33	24.73
*Allosaurus* average	23.00	4	12	0.82	6.89	88.14

**Note:**

Standard deviations of VEIW based on D’Emic et al. (2019) and the relative standard deviation based on *Alligator* (this study) are compared for *Majungasaurus*, *Ceratosaurus*, and *Allosaurus*. # transects and # measured VEIW are the numbers of transects and VEIWs measured in those transects to derive the mean VEIW and its standard deviation. Difference old–new VEIW SD (%) denotes how much the previously reported and the *Alligator* based SD differ (((old VEIW SD/*Alligator* VEIW SD) − 1) * 100; no difference would be 0%). SD, standard deviation; VEIW, von Ebner line increment width.

**Table 7:** Impact of VEIW ± 1SD on tooth formation time and replacement rate of sauropods.

Taxon	Tooth position	Mean tooth formation time (days)	TFT + SD (days)	TFT − SD (days)	TRR (count) (days)	TRR max-max (days)	TRR min-min (days)	TRR min-max (days)	TRR max-min (days)	Source
*Diplodocus* YPM 4677	I	187–183	264.0421	142.3784						D’Emic et al. (2013)
pmx	II	178–145	230.5016	124.2925	36	51.3812	27.7061	−67.3231	146.4104	
Average for tooth postion	III	144–113	189.3491	102.1020	33	47.0994	25.3972	−47.9298	120.4264	
	IV	110	156.9980	84.6574						
*Camarasaurus* UMNH 5527	I	130–113	173.4114	93.5080						
pmx	II	315–208	373.2271	201.2538	62	88.4898	47.7160	−118.6673	254.8731	
Average for tooth position	III	253	361.0954	194.7121	63	89.9170	48.4856	−76.4663	214.8689	
	IV	190	271.1784	146.2265	60	85.6353	46.1768	−39.3166	171.1287	
*Tornieria aficana* MB.R.2345	I									Sattler (2014)
mx sin	II	135.23	193.0124	104.0774						
Average for tooth position	III	148.93	212.5539	114.6146						
MB.R.2346	I	156.325	223.1156	120.3098						
pmx sin	II	118.60	169.2724	91.2761	37.73	53.8432	29.0337	−48.9626	131.8395	
	III	102.67	146.5315	79.0136	17.95	25.6192	13.8146	−56.5860	96.0197	
MB.R.2343	II	125.78	179.5130	96.7981	36.88	52.6300	28.3795	−54.3354	135.3449	
pmx dext	III	96.40	137.5874	74.1907						
*Giraffatitan brancai*	I	216.34	308.7749	166.4995						Kosch (2014)
MB.R.2180.1	II	229.59	327.6775	176.6923	73.46	104.8392	56.5320	−47.6400	209.0113	
pmx sin	III	158.40	226.0808	121.90868						
MB.R.2181.1	I	286.52	408.9365	220.5093	91.43	130.4938	70.3657	−57.9334	258.7929	
pmx sin	II	202.47	288.9814	155.8263	64.14	91.5460	49.3640	−41.6091	182.5190	
	III	138.33	197.4354	106.46234						
MB.R.2181.2	I	279.68	399.1709	215.2434	58.55	83.5611	45.0584	−100.3664	228.9859	
pmx dext	II	221.13.67	315.6098	170.1851	89.17	127.2206	68.6284	−27.3426	223.2431	
	III	145.93	208.2822	112.3113						
MB.R.2180.2	I									
mx sin	II	205.04	292.6484	157.8037	92.53	132.0642	71.2125	−16.1540	219.4306	
first 5 alveoli	III	132.85	189.6081	102.2416						
MB.R.2180.3	I	132.85	189.6081	102.2416						
mx dext	II	178.01	254.0630	136.9974	80.68	115.1522	62.0931	−20.2138	197.4591	
first 5 alveoli	III	139.79	199.5125	107.5824						
MB.R.2181.4	I	132.8481	189.6081	102.2416						
mx sin	II	178.01	254.0630	136.9974	80.68	115.1522	62.0931	−20.2138	197.4591	
first 5 alveoli	III	139.79	199.5125	107.5824						
MB.R.2181.3	I	211.37								
mx dext	II	224.10	319.8521	172.4726	74.07	105.7133	57.0034	−40.6182	203.3349	
first 5 alveoli	III	146.14	208.5777	112.4705						
MB.R.2180.14	I									
dent sin	II	227.89	325.2511	175.3839	114.20	162.9986	87.8931	−6.7549	257.6466	
first 5 alveoli	III	143.92	205.4110	110.7630						
MB.R.2180.13	I									
dent dext	II	236.83	338.0197	182.2691	104.14	148.6316	80.1461	−19.3872	248.1649	
first 5 alveoli	III	154.93	221.1250	119.2364						
*Brachiosaurus* sp. USNM 5730	I	280	399.8147	215.5906	52.88	75.4768	40.6999065	−108.7473	224.9232	D’Emic & Carrano (2019)
mx	II	260–227	350.6864	189.0993	97.59	139.2908	75.1092	−28.3666	242.7667	
Average for tooth postion	III	162–153	224.5698	121.0939						
dent	I	258–242	358.5753	193.3532	83.72	119.4828	64.4283	−45.7393	229.6503	
Average for tooth postion	II	185–150	239.0925	128.9249						
*Dicraeosaurus hansemanni*	I									Kosch (2010), Schwarz et al. (2015)
MB.R.2337	II	188.50	269.0375	145.0721	20.33	29.0208	15.6488	−95.9310	140.6007	
pmx dext	III	174.00	248.3423	133.9127	34.67	49.4782	26.6799	−62.1017	138.2598	
	IV	141.00	201.2429	108.5155	60.33	86.1110	46.4333	−2.6706	135.2149	
	V	74.75	106.6873	57.5286	76.00	108.4714	58.4906	39.4190	127.5430	
	VI	28.50	40.6768	21.9340						
MB.R.2338	I									
pmx sin	II	185.25	264.3989	142.5708	19.00	27.1178	14.6226	−96.8476	138.5881	
	III	175.25	250.1264	134.8747	54.00	77.0717	41.5591	−38.2347	156.8656	
	IV	123.25	175.9091	94.8548	50.67	72.3142	38.9937	−7.4796	118.7876	
	V	65.25	93.1284	50.2173	77.00	109.8986	59.2602	38.2157	130.9431	
	VI	32.00	45.6721	24.6276						
MB.R.2339	I									
pmx sin	II	189.00	269.7511	145.4569	10.00	12.8453	6.9265	−111.7778	131.5496	
	III	181.75	259.4035	139.8772	43.25	61.7288	33.2858	−57.7976	152.8121	
	IV	138.50	197.6748	106.5914	58.25	83.1376	44.8300	−7.9458	135.9133	
	V	80.25	114.5372	61.7615	56.50	80.6399	43.4831	28.3574	95.7656	
	VI	23.00	32.8269	17.7011						
MB.R.2336	I									
max dext	II	176.00	251.1968	135.4519	35.25	50.3107	27.1289	−65.4342	142.8738	
first 5 alveoli	III	149.00	212.6609	114.6724	62.75	89.5602	48.2932	−14.1827	152.0362	
	IV	95.00	135.5892	73.1132	55.25	78.8558	42.5211	16.3799	104.9971	
	V	38.60	55.0920	29.7071						
MB.R.2371	I									
dent sin	II	153.40	218.9409	118.0587	49.60	70.7918	38.1728	−30.0904	139.0550	
first 5 alveoli	III	103.80	148.1490	79.8858	61.60	87.9189	47.4082	19.6557	115.6714	
	IV	42.20	60.2301	32.4777						

**Note:**

Ranges of mean tooth formation times (TFTs) for tooth positions of the sauropods *Diplodocus* sp., *Camarasaurus* sp., *Torneria africana*, *Giraffatitan brancai*, *Brachiosaurus* sp., and *Dicraeosaurus hansemanni* and resultant tooth replacement rates (TRR) based on TFT from VEIW ± 1SD. D’Emic et al. (2013), D’Emic & Carrano (2019) only give TFT for some teeth, so the full range of TFTs found in the position is displayed, for other tooth positions the mean TFT found in this position in the jaw element is displayed. For explanation of TRR, TRR max-max, TRR min-min, TRR min-max, TRR max-min see methods. SD, standard deviation.

## Discussion

### A standardized, best practice protocol for calculating tooth ages

Based on our sampling tests, we propose the following best practices for calculating tooth ages, applicable to both extinct and extant taxa. (1) If possible, obtain data on VEIWs from synchrotron scanning or histological sectioning (this approach is preferred over estimates of VEIW obtained from a transfer function). (2) Measure CAH from the tip of the pulp cavity to the crown apex for each tooth in the dentition, via models obtained from CT scanning prior to preparation of histological sections. This approach is preferable as it is difficult to obtain a section from exactly the plane that contains both the crown apex and the tip of the pulp cavity. (3) Produce histological sections along the central axis perpendicular to von Ebner lines. (4) Measure VEIW between all visible von Ebner lines along the central axis, avoiding subsampling. This protocol presents a challenge as von Ebner lines in this region have the least optical contrast (Ricart et al. (2019), this study, M. D’Emic, 2018, personal communication) and are not always visible. If measuring von Ebner line along the central axis is not possible and transects must be taken in an alternate location, VEIW should be calculated perpendicular to von Ebner line orientation as near to the central axis as possible, and CAH measured along the true central axis used to calculate tooth formation time. Precise measurement of the CAH is important as it significantly influences calculation of tooth formation times and replacement rates. (5) SD from the average VEIW should be used to incorporate uncertainty into estimates. Our analyses indicate increasing similarity of the relative standard deviations derived in this and previous studies with increasing sample size, suggesting convergence on a confident measure of error that is widely applicable to archosaur datasets. Specifically, our relative standard deviation (derived from the mean standard deviation values of 87 individual transects with 4,654 VEIWs) most closely approximates relative standard deviations of *Majungasaurus* from D’Emic et al. (2019) (which was based on 54 transects with 544 VEIWs), and differs most from the relative standard deviation of *Allosaurus*, which was calculated using only 4 transects with 12 VEIWs (D’Emic et al., 2019) (Table 6). This suggests that applying the relative standard deviation of *Alligator* we provide herein is a better approach for generating confidence intervals around mean VEIW for taxa in which only a limited number of VEIW can be directly measured, than calculating SD from such a small number of measured VEIW. In addition, the *Alligator* based relative standard deviation can be applied to tooth formation times calculated based on a transfer function like the transfer functions for tooth length to tooth formation time form D’Emic et al. (2013, 2019). We advise to take the intradental VEIW variability we document here into account also when calculating and discussing tooth replacement rates. This can be done by comparing those based on minimum and maximum tooth formation times (−23.04% and +42.73% in our sample respectively) for both adjacent tooth positions to the calculated baseline value. This introduces a margin of error to reports of tooth formation time and replacement rate. (6) In homodont to weakly heterodont taxa, researchers can be confident in applying mean VEIWs derived from transects perpendicular to von Ebner lines to derive calculations of tooth formation time and replacement rate in other regions of the tooth rows, and despite growth stage.

### Tooth geometry is more impactful on VEIW than growth

Currently our data do not support a straightforward trend in VEIW variation that can be linked to tooth growth over time. Within our sample, significant differences in mean VEIW occur between the apicalmost and thus oldest subsection of the tooth central axis and sections grown later. Therefore it is tempting to suggest that subsampling VEIW either in the apicalmost or basalmost region of the tooth will lead to over or underestimates of tooth formation time respectively. However, our sample contains a few replacement teeth (equivalents to the oldest and most apical crown parts of more mature teeth) that have wider mean VEIWs than the functional teeth in their respective alveoli (Table 7), which is at odds with the pattern we document of thinner VEIWs in the apical area. Moreover, when we compare multiple teeth of the same individual, we observe consistent patterns in VEIW that suggest external factors are influencing VEIW similarly within all teeth of the dentition. These conflicting data suggest the crowded von Ebner lines we observe in our sample are not reflective of a general pattern related to tooth growth that is consistent across all individuals, but rather is likely related to changing levels of metabolic activity of the individual we sampled during tooth growth. Such an interpretation fits with other studies documenting variations in metabolic activity to tooth formation time and replacement rate and thus daily tooth growth documented by VEIW across a wide array of tetrapods including squamates (*Anguis fragilis*, *Chalcides sexlineatus*, and *Chalcides viridanus*) (Cooper, 1966; Delgado, Davit-Beal & Sire, 2003) and mammals (Klevezal, 1996: 66f). Future research into the underlying factors of tooth growth under controlled conditions (Wu et al., 2013) might provide more conclusive explanations for actual intradental VEIW variation. Thus, we caution that researchers should not interpret the pattern we document here of more crowded von Ebner lines early in tooth growth as applicable widely to *Alligator* or transferable to other archosaurs. What the data on subsampling do suggest is that subsampling VEIW anywhere along the central axis of the crown, as opposed to measuring VEIW along the entire central axis can impact tooth formation time calculation by up to 12%. We deem this an acceptable deviation as it lies within the margin of error created by applying VEIW ± 1SD (grand mean), but note that the practice of sampling all visible VIEWs reduces this variation and should be preferred.

Variation in VEIW and observed von Ebner lines evident along transects off the central axis are more a function of tooth geometry than tooth growth and can have more extreme effects on calculations of tooth formation time and tooth replacement rate. We document that tooth formation times derived from mean VEIW of all von Ebner lines along transects lateral to the pulp cavity only capture about 83% of the age represented by the von Ebner lines along the CAH. Reasons for this are not only that fewer discernible von Ebner lines are represented along such transects, but also that VEIW decreases in lateral transects by more than a third due to tooth shape. Measuring VEIW as close to the central axis as possible mitigates problems introduced by other transect orientations and should be preferred over other methods such as measuring VEIWs in multiple transverse sections of the same tooth (Erickson, 1992), particularly in high-crowned taxa. Mean VEIWs obtained by multiple transverse sections or a zig-zag pattern (Fig. 1B in File S1) systematically underestimate the mean VEIW by conflating VEIWs of varying distance lateral of the pulp cavity, however, they are to be favored compared to transects that introduce error by measuring VEIW oblique to the von Ebner lines. Future research can make attempts to expand the dataset for crocodylians to include species with varying tooth geometry, including globidont taxa to see how tooth shape impacts VEIW and if different potential error margins have to be used when utilizing VEIW measurements in teeth with morphology distinct from our *Alligator mississippienisis* sample.

### Ontogeny and VEIW

Investigating ontogenetic sampling effects on mean VEIW is hampered by the small sample size of this study, but the ontogenetic development of dental organization we find differs from previously reported patterns (Edmund, 1962; Erickson, 1992; Hanai & Tsuihiji, 2018). Erickson (1992) found a significant relationship between mean VEIW and body length in *Alligator*; however, we find no such strong evidence for ontogenetic sampling effects on mean VEIW. When we combine our data with that of Erickson’s (1992, 1996a) data, the relationship between mean VEIW and body length does not reach a level of 95% of significance. However, we note that a caveat of using the specimen level mean VEIWs from Erickson (1992, 1996a) is that his VEIWs were derived from a “zig-zag” pattern of measurement (Fig. 1B in Fig. S1) (i.e., reported mean VEIWs combine VEIWs from near the central axis and marginal measurements). Our data indicate that this technique leads to a systematic underestimation of VEIW compared to our central axis-based measurements. Preliminary corrections for reported VEIWs of Erickson (1992, 1996a) by adding 26.667% to the reported width (following the assumption that 1/5 of the transects used by Erickson are using VEIWs that are 33% too short) do increase the correlation of body length to specimen mean VEIW to a significant value with a better fit for a quadratic regression (reduced major axis regression *p* = 0.0455, *R*^2^ = 0.457; *R*^2^ = 0.680 for a quadratic regression) (Fig. 6 in File S1; Table 1). However, we do not deem this ad hoc correction of Erickson (1992, 1996a) mean VEIWs precise enough to confidently apply it to all of his measurements obtained from Erickson’s zig-zag counting and measuring method (e.g., in Table 5).

It is unclear if the addition of more data or a more reliable method to account for the systematically underestimated mean VEIWs of Erickson (1992, 1996a), will exacerbate the lack of correlation, or will more conclusively link mean VEIW and ontogenetic status. The former is consistent with the mostly small and non-systematic variation in mean VEIW of hadrosaur and theropod dinosaur species of different ontogenetic status reported in Erickson (1996b) and the constant VEIW in a juvenile and adult baurusuchid crocodylian reported by Ricart et al. (2019). As of now, our data cannot confidently support a simple trend of increasing VEIW during ontogeny that is strong enough to produce significant differences (α = 93% or more) in the mean VEIWs of our sampled teeth, but do suggest a slight ontogenetic increase in specimen mean VEIW. Due to the lack of significance of both of our statistical tests our preliminary assessment is that VEIW does not change systematically and uniformly during ontogeny, making it possible to apply mean VEIWs obtained to various ontogenetic stages of a species without introducing an ontogenetic scaling factor.

Although we find no substantive impact on mean VIEW across ontogeny, other research indicates that tooth formation time and tooth replacement rate—and thus tooth longevity—do increase ontogenetically (File S1, Erickson, 1992, 1996a). Therefore other factors related to tooth size and jaw size likely account for this observation. These could include size increase of new teeth before eruption/replacement in later tooth generations, more space for replacement teeth in the alveolar ramus/alveoli/dental crypts, longer time required to dissolve the roots of larger teeth before they are shed, and/or more space in the pulp cavity/close to the functional tooth. Such a trend is supported by allometric tooth size increase compared to skull length reported by Brown et al. (2015).

Our study uses data from three individuals at different ontogenetic stages to reconstruct ontogenetic changes and growth patterns that are hypothesized to occur in a single individual in a similar manner—a method used often in paleontology (Erickson, 2014)—in contrast to longitudinal studies that chronicle ontogenetic trajectories of individuals over time. No longitudinal study measuring VEIW have been carried out for archosaurs to date; Edmund (1962) did not measure VEIWs and Erickson (1992, 1996a) did rear alligators for labeling studies of von Ebner lines, but used them as single data points for VEIW.

## Conclusions

Our results indicate that the greatest challenges to accurately estimating tooth formation time and calculating tooth replacement rate for extinct taxa rests in (1) generating a transect perpendicular to von Ebner line orientation for the measurement of VEIW (oblique measurements reduce tooth formation time estimates by up to a third in *Alligator*); and (2) measuring VEIW and crown height along a transect that bisects the central axis as opposed to other regions of the tooth. VEIW decreases with increasing distance from the central axis (up to 36%) and use of mean VEIW values from these regions will produce inflated tooth formation times; the greater the crown height, the more intense the impact errors in calculations of mean VEIW. Measuring VEIW along the central axis also helps to keep transect orientation perpendicular to von Ebner lines.

We find that transects misaligned with von Ebner line orientation or with respect to the central axis impact the calculation of mean VEIW far more than the acts of subsampling within transects, applying mean VEIW from one tooth in the tooth row to crown axis height of another tooth, and ontogenetic status of the individual sampled. We document up to 12% variation in calculations of mean VEIW when derived from subsampling the central axis transect and note that variation in VEIW is likely an idiosyncratic pattern linked to organismal life history. Therefore, we cannot recommend any standardized subsampling location along the central axis and recommend directly measuring the distance between all visible von Ebner lines along to minimize error. However, we note that subsampling error is within the margin of error of VEIW ± 1SD derived and therefore if a VEIW cannot be measured from a complete transect it is sufficient to use a subset of measurements along a central axis transect to calculate a mean VEIW for a whole tooth. Intramandibular sampling effects do not significantly influence the mean VEIW as there is no systematic variation of mean VEIW associated with tooth position along the tooth row in our sample. It is sufficient to have a few samples from teeth of a dentition to make reliable prediction of the tooth formation time for other teeth, provided that the CAH is known.

According to our limited data, ontogenetic status does not significantly affect calculations of mean VEIW (below a confidence value of α = 93%); therefore, we preliminarily suggest that a mean VEIW obtained for a specimen of a taxon can be applied to the dentition of other specimens regardless of growth stage. However, we note that this factor warrants more study as ad hoc corrections of data for younger specimens of *Alligator* suggests the relationships might reach a higher degree of significance.

## Supplemental Information

10.7717/peerj.9918/supp-1 Supplemental Information 1 Additional background and figures for transect orientation, ontogenetic scaling of VEIW, the relationship of TFT to TH and CAH, and scaling of Tooth replacement rate to body length.

10.7717/peerj.9918/supp-2 Supplemental Information 2 Table and Figure TFT+-SD tooth specific.

10.7717/peerj.9918/supp-3 Supplemental Information 3 VEIW according to tooth position with ANOVA. Mean VEIW for the sampled tooth positions in mm. Only measurements with the same transect orientation were used (central axis, excerpt for ’ root base; * a combination of root base and crown base transects). Some tooth positions were sampled but had a different transect orientation from the other teeth and were excluded from table and analyses. Second generation replacement teeth (one in the dentition of the smallest specimen, four in the dentition of the medium sized specimen, three in the dentition of the largest specimen) are excluded. ANOVA result table for the three specimens. ANOVAs based on VEIWs of all sampled alveoli with a central axis orientation and of just those alveoli with three or more central axis samples below the table, using its values.

10.7717/peerj.9918/supp-4 Supplemental Information 4 Impacts Assessments of Tooth Growth and Replacement Rates Calculations in Archosaurs. Raw data of tooth length (TH) and central axis height (CAH; measured from the tip of the pulp cavity to the crown apex) is provided in supplementary file Data S1. It contains raw TH and CAH as well as TH and CAH corrected for average enamel thickness. Data S1 also contains the calculations used for all tables, graphs and statistic tests performed mentioned in this work, including the computation of average enamel thickness for all individuals.

10.7717/peerj.9918/supp-5 Supplemental Information 5 Small alligator NCSM 100803 all Ebener lines. The raw measurements of von Ebener line increment widths (VEIWs) are provided in Data S2, S3, and S4. The data shows all measured transects and next to the name of the picture file it was measured from the position of the transect within the tooth. On a second sheet in each of these files tooth formation times (TFT) are calculated for scenarios using CAH, TH and transect length for each transect.

10.7717/peerj.9918/supp-6 Supplemental Information 6 Med alligator NCSM 100804 all Ebener lines. The raw measurements of von Ebener line increment widths (VEIWs) are provided in Data S2, S3, and S4. The data shows all measured transects and next to the name of the picture file it was measured from the position of the transect within the tooth. On a second sheet in each of these files tooth formation times (TFT) are calculated for scenarios using CAH, TH and transect length for each transect.

10.7717/peerj.9918/supp-7 Supplemental Information 7 Large alligator NCSM 100805 all Ebener lines. The raw measurements of von Ebener line increment widths (VEIWs) are provided in Data S2, S3, and S4. The data shows all measured transects and next to the name of the picture file it was measured from the position of the transect within the tooth. On a second sheet in each of these files tooth formation times (TFT) are calculated for scenarios using CAH, TH and transect length for each transect.

## Abbreviations

CA: central axis
CAH: central axis height
Dent: dentary tooth position
max: maximum tooth formation time estimate
min: minimum tooth formation time estimate
Mx: maxillary tooth position
NCSM: North Carolina Museum of Natural Sciences
Pmx: premaxillary tooth position
SD: standard deviation
TFT: Tooth Formation Time
VEIW: von Ebner Increment Width between von Ebner Lines

## Additional Information and Declarations

### Data Availability

The following information was supplied regarding data availability: The raw measurements of von Ebener line increment widths (VEIWs) and the raw data of tooth length (TH) and central axis height (CAH; measured from the tip of the pulp cavity to the crown apex) are available as Supplemental Files.
