# Supplementary material for: Sampling impacts the assessment of tooth growth and replacement rates in archosaurs: implications for paleontological studies
Source: PeerJ. 2020 Sep 18;8:e9918. doi: 10.7717/peerj.9918 (PMC7505082; doi:10.7717/peerj.9918)
Supplement: Supplemental Information 1 [file peerj-08-9918-s001.pdf]

Sampling Impacts the Assessment of Tooth Growth and Replacement Rates in Archosaurs: Implications for Paleontological Studies, Supplemental 1

Kosch, Jens[1,2]
Zanno, Lindsay[1,2]

1. Department of Biological Sciences, North Carolina State University, Raleigh, NC, USA
2. Paleontology, North Carolina Museum of Natural Sciences, Raleigh, NC, USA

Transect orientation and the effects of sampling location for on VEIW measured in a tooth
We refute the assumption that mean von Ebner increment width (**VEIW**) is consistent regardless of the transect position used for sampling. There are two aspects to the effect of transect orientation: 1) transects oriented perpendicular to von Ebner lines (**VELs)** but variable in tooth location (with regard to the tooth from root to apex), and 2) transect orientation relative to von Ebner trendlines (perpendicular or obliquely oriented).

With regard to location on the tooth, sections that do not precisely section the central axis (**CA**) do not preserve all von Ebner lines. Figure 1 of Erickson (1992) (redrawn in Fig. 1b) and Figure 2 of the main text illustrate sections through the CA. Transverse sections omit von Ebener lines, particularly those of the crown apex (Fig. 1a). Von Ebner line counts along mesiodistal or labiolingual transects (lateral to the pulp cavity) are consistently fewer than those derived from a CA transect (upwards of 40%). Erickson (1992) outlines a method to avoid missing those lines in pure line counts, by tracing the last clearly visible von Ebner line further to the crown apex and starting a new transect from that von Ebner line (which can be repeated as many times as necessary to record all von Ebner lines of the tooth). The zig-zag pattern following the last visible von Ebner line to a further apical position allows to count all von Ebner line in the tooth (Fig. 1b)

However, this method does not consistently record VEIWs of the same relative position, so mean VEIW obtained from these counts (as done by Erickson [1992]) will be diminishing the further laterally from the CA the von Ebner lines are positioned (see the distance between duplicated von Ebner lines crossed in the basal and apical transect in Fig. 1). We find that mean VEIW in a tooth decreases laterally (Fig. 1, Fig. 2) even when sampled perpendicular to von Ebner trendlines; VEIWs are thickest along the CA from above the pulp cavity to the crown apex and thinnest when taken as true transverse planes near the enamel dentin junction. Transects along the CA yield a more constant VEIW due to not measuring the laterally decreasing width of VEIW while also keeping the transect perpendicular to the von Ebner lines (Fig. 1a).

However, variation in VEIW is less affected by transect location as it is by transect orientation relative to von Ebner trendlines. Transects that cross von Ebner lines obliquely (Fig. 3) (e.g., top of the

pulp cavity to the enamel dentin junction, in areas adjacent to the apex (Fig. 3b in the main text) yield VEIWs up to nearly twice the width than those made perpendicular to von Ebner trendlines (this is average from some test measurements [Fig. 4], the exact value depends on the orientation [angle] of the trendline to the von Ebner lines; see also table 2 in the main text). This variation results in differential calculations of TFT depending on transect orientation.

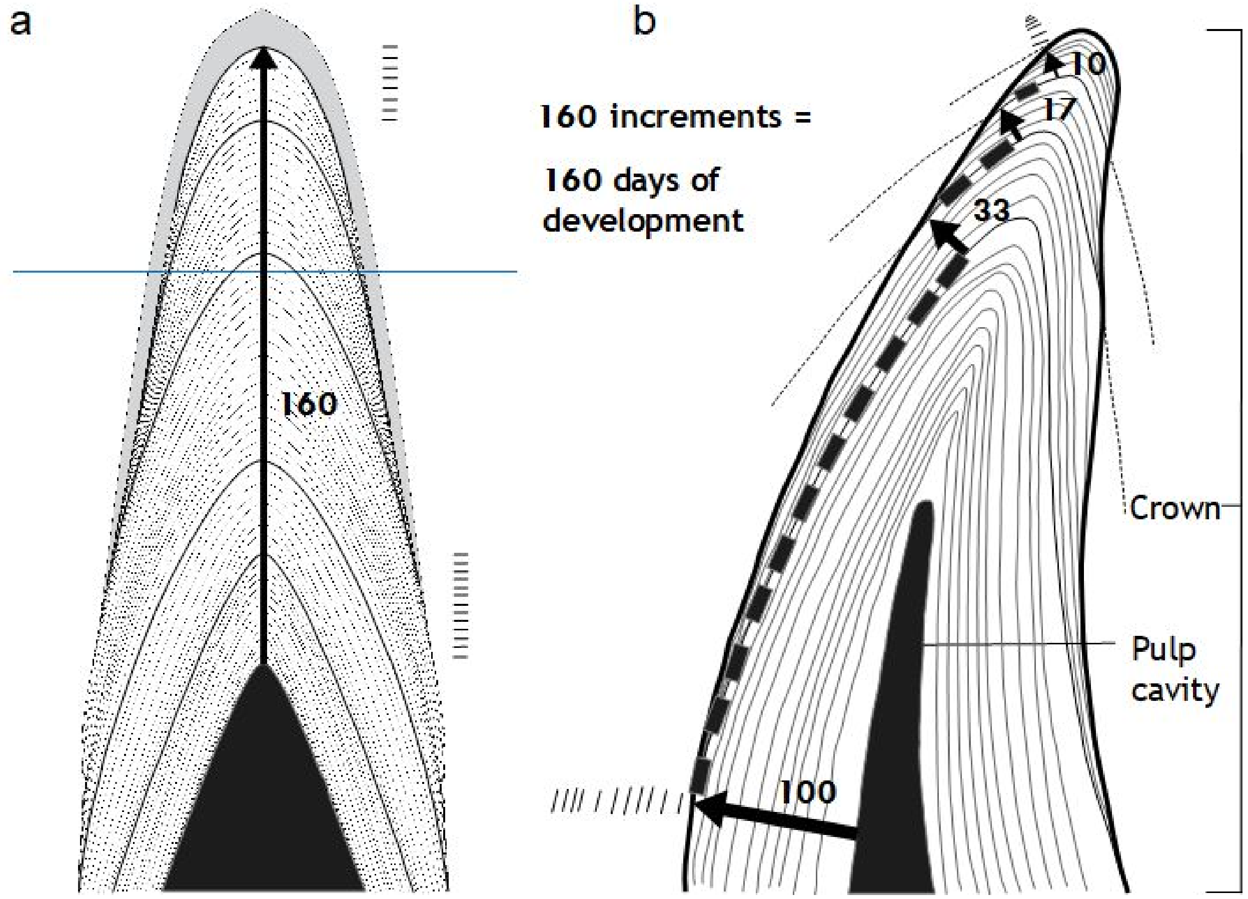

Fig. 1 Different schemes for transects to count von Ebner lines and influence on VEIW
Areas with arrows are the transects. von Ebner line of the lowest and the topmost sections are duplicated outside thee tooth. **a** Transect through CA, counting all von Ebner line in one transect (arbitrary set to 160, as in Erickson [1992] Fig. 1) with a more constant VEIW due to not measuring the laterally decreasing width of VEIW while also keeping the transect perpendicular to the von Ebner line. **b** Figure 1 from Erickson (1992) redrawn with minor changes in von Ebner line depiction.

Ontogenetic scaling of VEIW

When we derive mean VEIW across the whole dentition of our sampled individuals (from smallest to largest: NCSM 100803, NCSM 100804, and NCSM 100805), we find a slight increase during ontogeny (Fig.5a); however, three individuals are not enough for a meaningful statistic comparison. To our data, we added the mean VEIW of differently sized *Alligator* specimens ranging in body length from 0.60 m to 3.20 m from Erickson (1992 and 1996) (Fig. 5b). We find the relationship between VEIW and body length does not reach 95% significance ($p = 0.0738$) but find an ontogentic signal ($R^2 = 0.3889$). Thus, it

seems likely that there is no reliable trend of increasing VEIW during ontogeny, that needs to be taken into account when applying mean VEIW across growth series to derive TFT. Erickson 1996 adds data points for tooth replacement rate of hatchling *Alligator* to his Fig. 3.

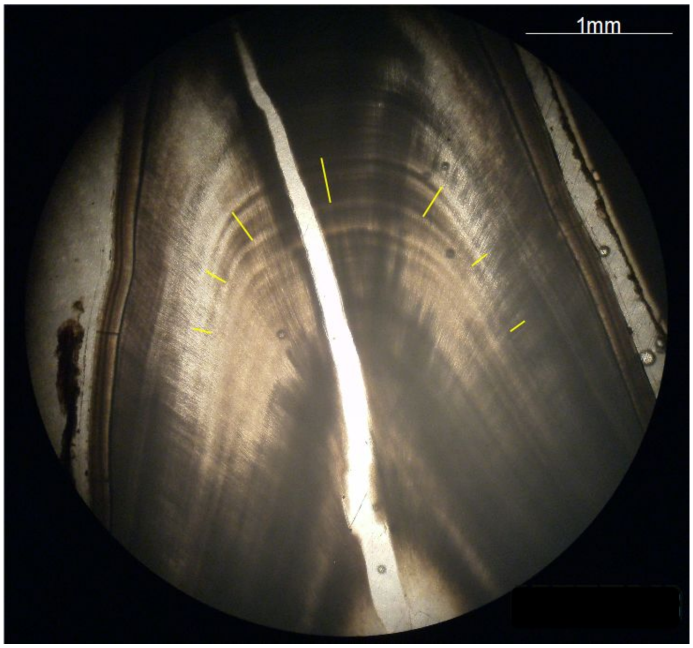

Fig. 2 Variation of Increment Line Thickness within a Tooth
Yellow lines mark the same group of incremental growth lines. The height of these lines is decreasing lateral to the CA.

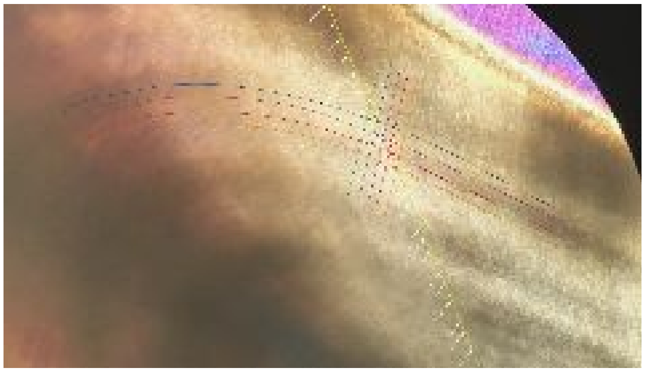

Fig. 3 VEIW in oblique transects
The straight Yellow line is the transect. The individual VEL are marked with arrows where they intersect with the transect. Select neighboring VEL are accentuated by blue, purple, red, and organge lines. Lines in the same colors and green are showing an orientation perpendicular to the VEL. Sections of the transect line crossing between the accentuated VELs are marked by the corresponding colors.

However, as outlined in the previous section, Erickson (1992, 1996) used a zig-zag method when counting VEIW, which leads to a systematic underestimation of mean VEIWs by conflating lateral VEIWs measured far from the CA with wider VEIWs measured closer to the CA.
We performed a preliminary correction for reported VEIWs of Erickson (1992, 1996) by adding 26.667% to the reported width (following the assumption that 1/5 of the transects used by Erickson are using VEIWs that are 33% too short). This does increase the correlation of body length to specimen mean VEIW to a more significant value (linear regression $p < 0.05$ [$p = 0.0455$], $R^2 = 0.457$; $R^2 = 0.680$ for a quadratic regression) (Fig. 6, table 1). However, we do not deem this ad hoc correction of Erickson's (1992, 1996) mean VEIWs precise enough to confidently apply it to all of his measurements obtained from Erickson's zig-zag counting and measuring method (e.g. in table 5 of the main text).
Our preliminary assessment is that VEIW does not change systematically during ontogeny, making it possible to apply mean VEIWs obtained to various ontogenetic stages of a species without introducing an ontogenetic scaling factor.

Tooth age by tooth and crown height
If all three individuals of different ontogenetic status are treated as a single sample there is a significant correlation between TFT calculated from VEIWs and central axis height (**CAH**) and tooth height (**TH**) [$p = 5.56291E-19$ and $p = 7.66367E-12$ respectively], but the coefficient of determination R^2 of the

corresponding linear regressions is low for the relationship with TH (0.6345 and 0.8175 respectively, see Fig. 7). If other types of function are used for TH even the best result (a function of the type $y = k x^a$) only explains 80.46% of the spread of the data (Fig. 8a). If the three individuals are treated separately (Fig. 8b), the small alligator's tooth age can be sufficiently described by various transfer functions (those with the highest coefficient of determination are displayed in Fig. 8b), but in the medium sized alligator only CAH shows a significant correlation to TFT (which is in part circular reasoning, as TFTs based on the crown height and tooth specific VEIWs) and the fit to the data spread is not very good ($R^2 = 0.49$ for a function of the type $y = k x^a$) and the large alligator has a significant relationship between tooth age and either tooth height ($R^2 = 0.73$, for an exponential function as an approximation) or crown height ($R^2 = 0.82$, for a quadratic function).
This lack of a fitting transfer function for the medium sized individual should serve as a caveat for constructing tooth-height-tooth-age relationships sensu D'Emic et al. (2013), based on lower quality VEIW measurements with few measured von Ebner line and multiple different orientations of the transect axis.

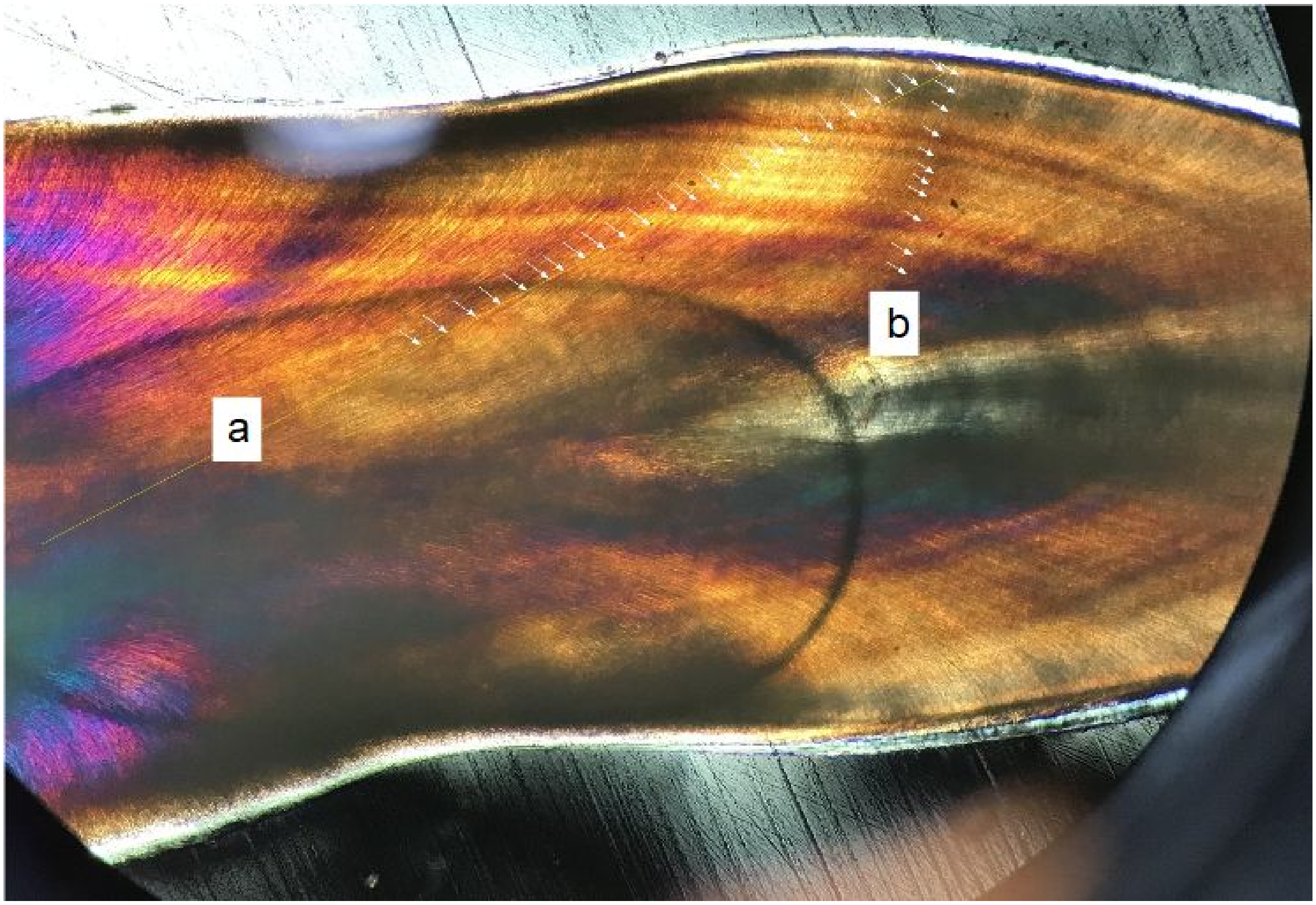

Fig. 4 Oblique transect orientation
The straight yellow line is the transect. Arrows denote the intersection of the von Ebner lines with the transect. Transect a is strongly oblique to the von Ebner line and reaches from the crown base near the pulp cavity to the mid-crown. Transect b meets the von Ebner line in perpendicular fashion, but includes only the von Ebner line a transverse section would have. From the first premaxillary alveolus of the medium sized alligator [NCSM 100804].

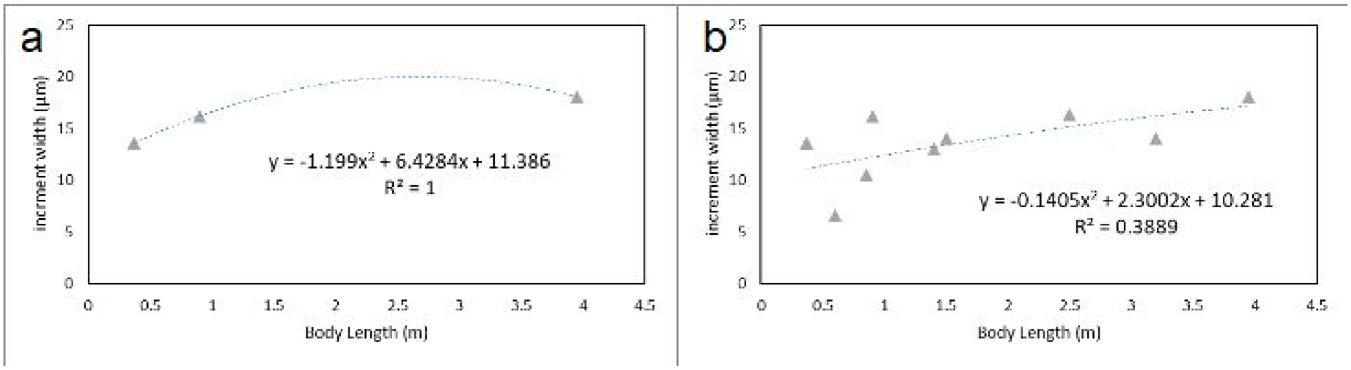

Fig. 5 Mean VEIW of individuals during ontogeny of *Alligator*
a Mean VEIW vs. body length for specimens in this study. **b** Mean VEIW vs. body length for this study and Erickson (1992, 1996). Both of the plots seem to suggest a slight increase of VEIW in ontogeny, but the plot with nine specimens establishes that the fit of the trend is not good ($R^2 = 0.39$) and the correlation for an linear regression is not significant ($p = 0.0738$)

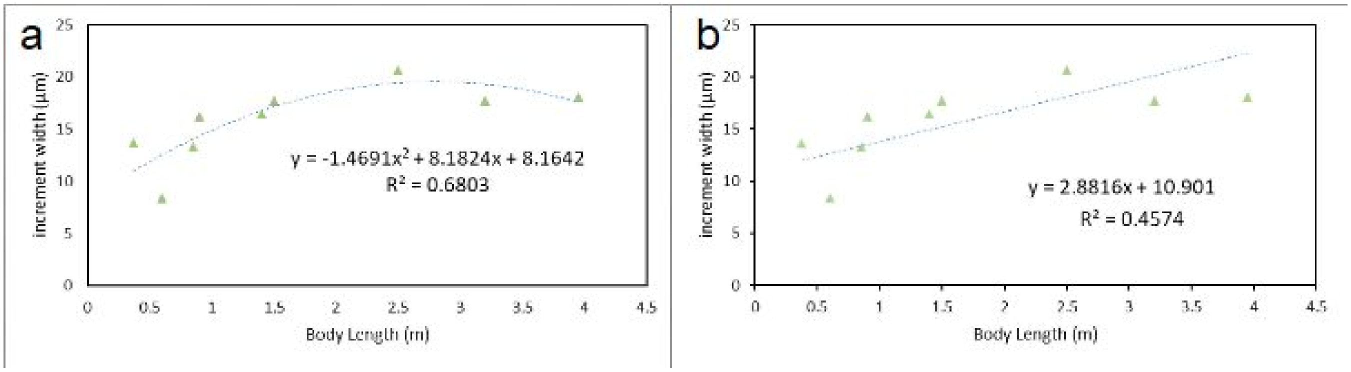

Fig. 6 Corrected mean VEIW of individuals during ontogeny of *Alligator*
a Corrected mean VEIW vs. body length for specimens in this study and Erickson (1992, 1996 with quadratic regression. **b** Corrected mean VEIW vs. body length for this study and Erickson (1992, 1996) with a reduced major axis regression. Both of the plots seem to suggest a slight increase of VEIW in ontogeny, but the coefficient of determination better fits a quadratic regression ($R^2 = 0.68$) than for the linear regression ($R^2 = 0.46$) and the correlation for an reduced major axis regression is significant ($p = 0.0455$).

Table 1 VEIW and tooth replacement rate in relationship to body size

Mean VEIW as reported by Erickson (1992, 1996) or obtained from our study. Mod. VEIW is reported VEIW multiplied by 1.2667 (see text for explanaion). P values are from an linear regression.

	Body length (m)	Sample size	Mean tooth replacement rate (days)	mean VEIW (µm)	mod. mean VEIW
This study	0.37	1	87.0467	13.6	13.6
Erickson 1996	0.6	2	83	6.6	8.36
Erickson 1996	0.85	2	109	10.5	13.3
This study	0.9	1	133.6184	16.2	16.2
Erickson 1996	1.4	2	122	13	16.467
Erickson 1992	1.5	?	115	14	17.733
Erickson 1992	2.5	?	154	16.3	20.647
Erickson 1992	3.2	?	260	14	17.733
This study	3.95	1	278.9975	18.07	18.07

	R^2	p value	mod. R^2	mod. p value
VEIW All data	0.387	0.0738	0.457	0.0455
VEIW Erickson 1996 & this study	0.057	0.698	0.36	0.2852
VEIW Erickson 1996	0.89	0.2157	0.89	0.2157
VEIW This study	0.787	0.3056	0.787	0.3056
replacement rate all alligators	0.902	9E-05	0.902	9E-05
replacement rate this study	0.991	0.0618	0.991	0.0618
replacement rate all + Erickson 1992	0.886	2E-05	0.886	2E-05
replacement rate all + Erickson 1996	0.896	1E-05	0.896	1E-05

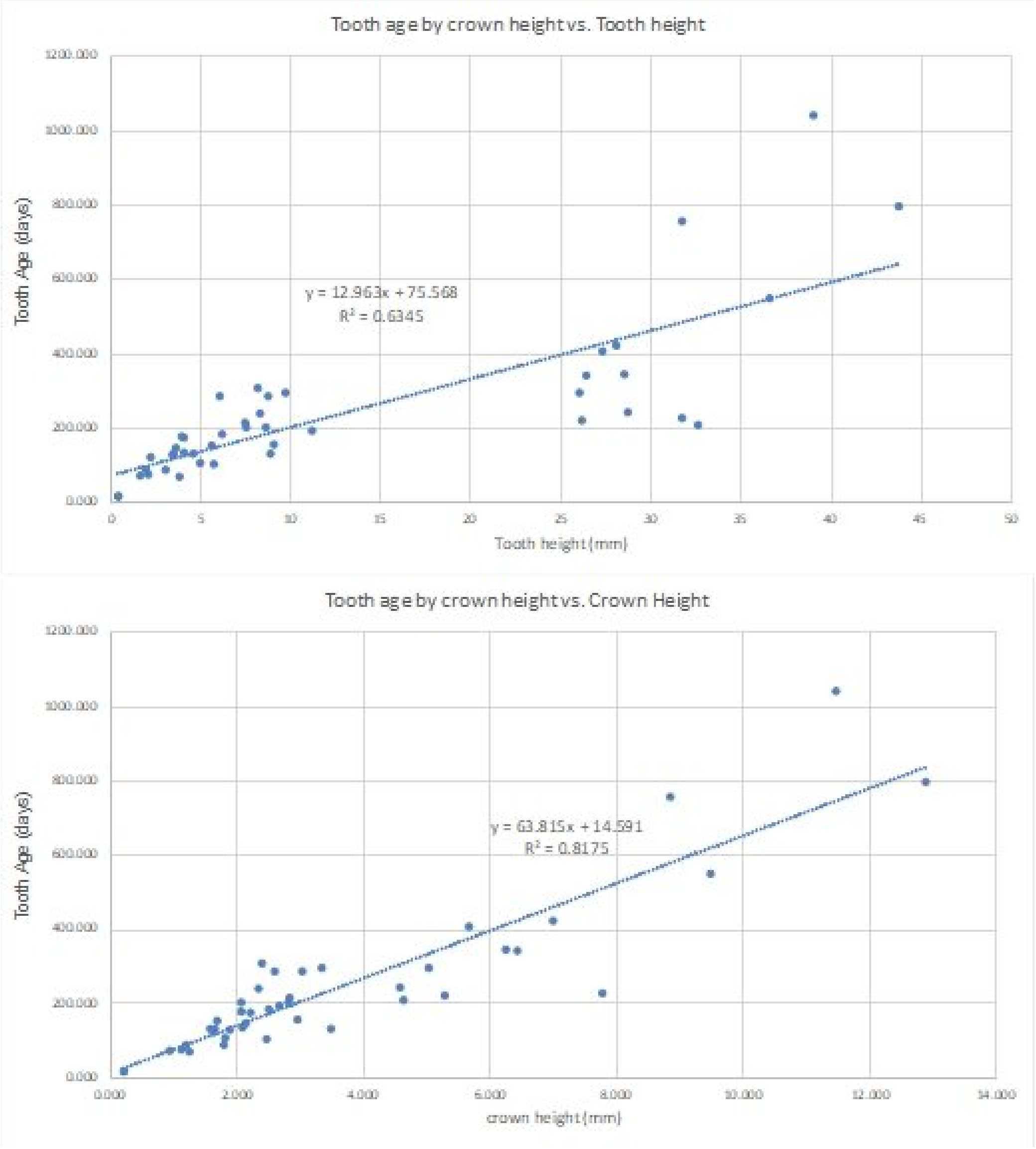

Fig. 7 Tooth age of *Alligator* to TH and CAH with linear trendlines

Tooth age derived from mean VEIW (= TFT) of individual teeth and CH plotted against TH and CAH with linear trendlines. All three *Alligator* specimens in the study are treated as one dataset for these.

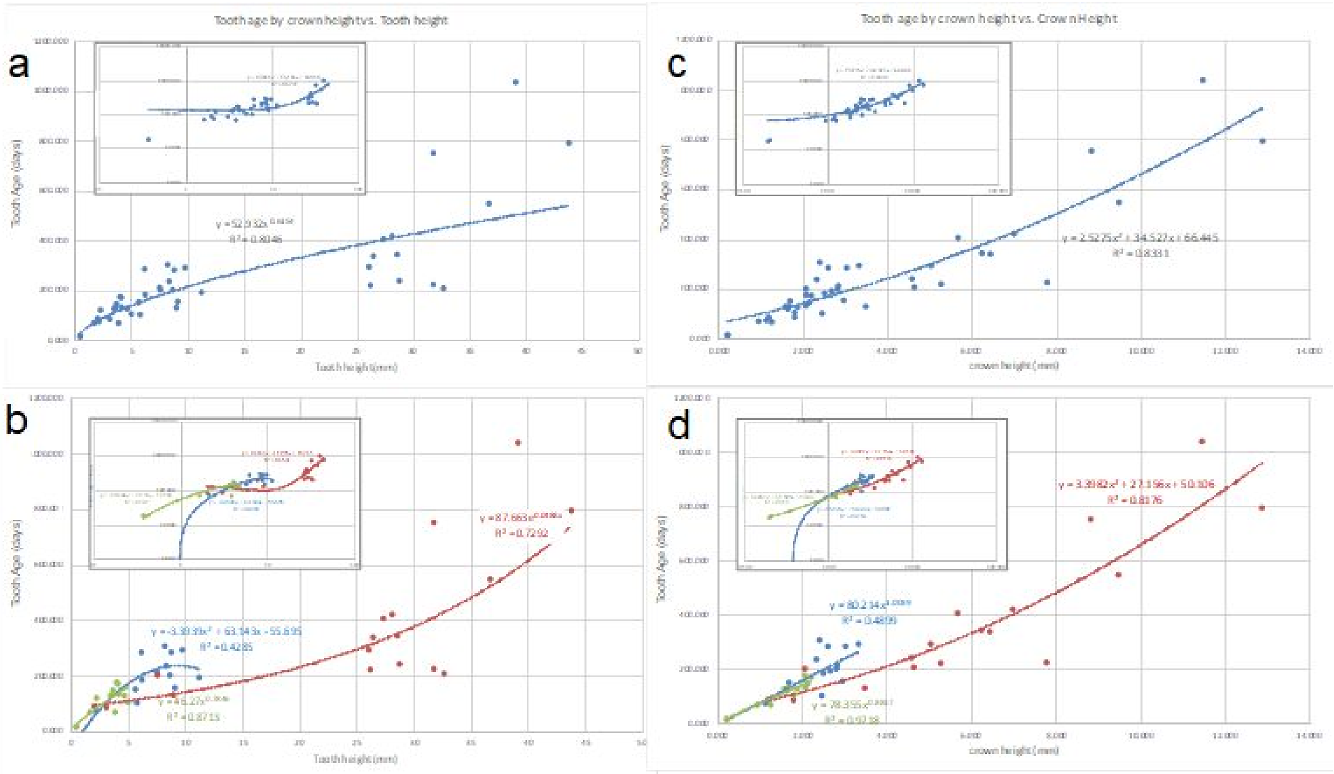

Fig. 8 TFT of *Alligator* to TH and CH with the best supported trends

a & **b** TH to TFT; **c** & **d** CAH to TFT. **b** & **c** show a treatment of the three specimens as three different datasets, **a** & **c** a treatment that treats all teeth as the same dataset. Small insets show the respective plots in a logarithmic scale and consistently use polynomes of the second order for the trendlines. **a** uses a function of the type $y = k x^a$. In **b** the small *Alligator* (NCSM 100803) uses a function of the type $y = k x^a$. The medium sized specimen (NCSM 100804) uses a polynome of the second order for the trendline. The large sized specimen (NCSM 100805) uses an exponential function for the trendline. **c** uses a polynome of the second order for the trendline. In **d** the small (NCSM 100803) and the medium sized specimen (NCSM 100804) use functions of the type $y = k x^a$. The large sized specimen (NCSM 100805) uses a polynome of the second order (= quadratic function) for the trendline.

Scaling of tooth replacement rate

Tooth replacement rate was derived from mean VEIW of the teeth and CAH, so it combines those observations, and when viewed in an ontogenetic context shows mainly the impact of tooth size (and thus CAH) increase.

When mean replacement rates for upper and lower dentition are observed it appears that mean central axis height (CAH) of the upper dentition is greater and replacement rate is slower in the medium (NCSM 100804) and large (NCSM 100805) *Alligator* (Fig. 9). It is also apparent this trend is much more pronounced in the large specimen. That this is more related to general trends of CAH (and thus TFT) than to differences in CAH of replacement teeth to CAH of functional teeth can be seen when the relations of the CAH are compared between the upper and lower dentition and the medium and large specimen. In the medium sized specimen the functional tooth's CAH is 3.02 times that of the replacement tooth's CAH

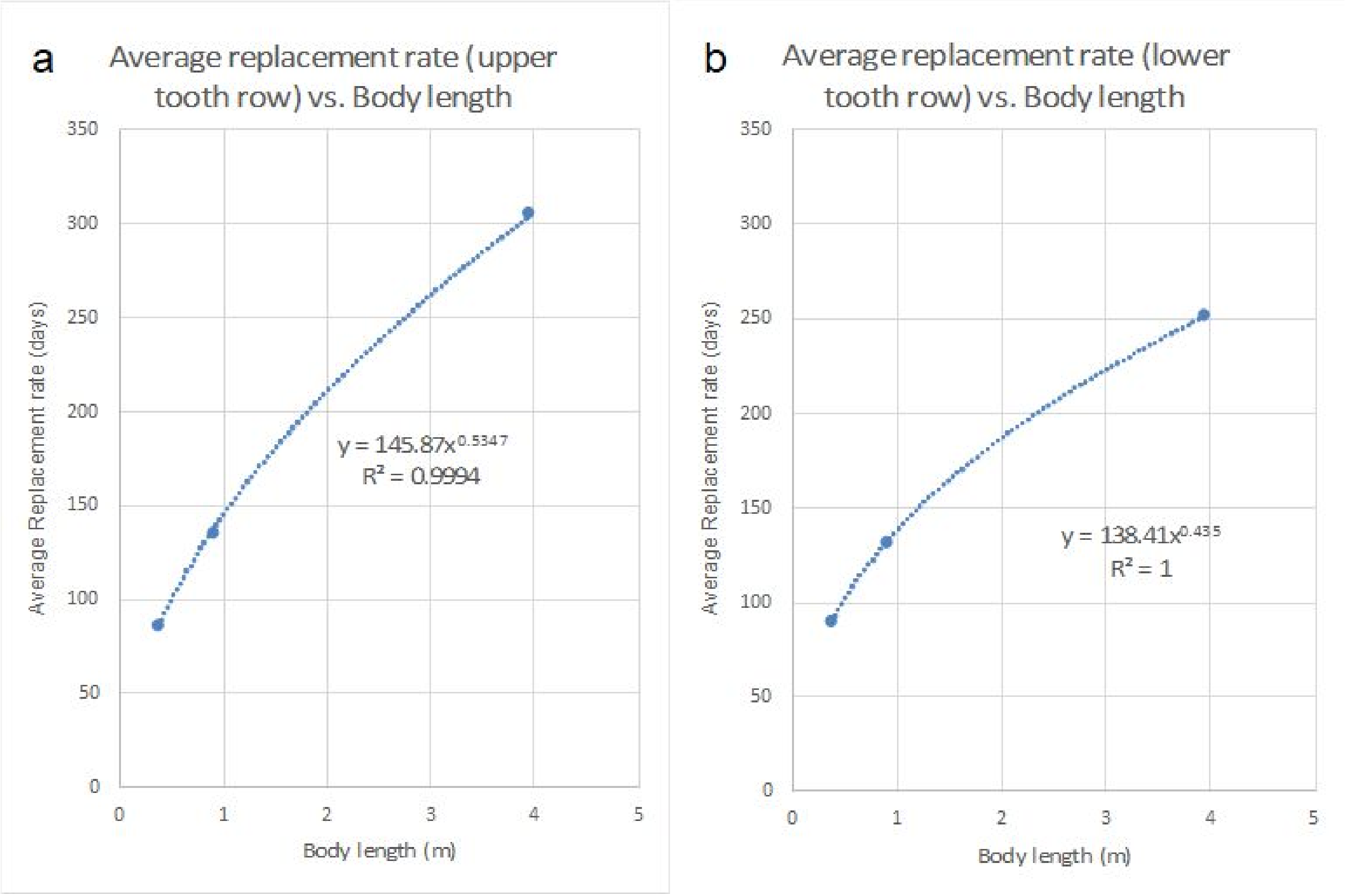

Fig. 9 Tooth Replacement Rate of upper and lower jaw of *Alligator* by Body length
a tooth replacement rate of the upper tooth row ; **b** tooth replacement rate of the lower tooth row on the left.

for the upper dentition and 3.07 for the lower dentition. For the large specimen the functional tooth's CAH is 2.66 times that of the replacement tooth's CAH for the upper dentition and 2.73 for the lower dentition. This is a relatively small difference compared to the more than 50 days difference of the average replacement rate in the upper and lower dentition of the large alligator in contrast to the less than 4 days difference of upper and lower replacement rate in the medium sized specimen. However, this perceived difference of upper and lower mandibles and the specimens might be warped by missing functional teeth (and perhaps also replacement teeth) of the large specimen (NCSM 100805). Pmx 4 and Mx 9 are missing in the upper jaw, whereas Dent 1, 3, 17, 18, 19, and 20 are missing in the lower jaw. Whether their presence would have changed this trend or even reinforced it is an open question. In the medium sized specimen the distalmost Dent teeth have comparatively less differences in CAH between functional and replacement teeth.
When those values are further combined to a specimen mean replacement rate the values can be compared to those derived from Erickson (1992). Erickson 1996 adds data points for tooth replacement rate of hatchling *Alligator* to his Fig. 3 and Erickson 1992 Fig. 13 does the same, both times referring to Westergaard and Ferguson 1990; but this source does not include any notes on the body length of the

individuals; thus, those hatchling individuals which in fact anchor the whole lower half of the linear regression were left out when we entered the additional data from Erickson (1992, 1996) in Fig. 10. Furthermore when we tried to derive body length and tooth replacement rate for these two individuals from Fig. 3 in Erickson 1996 and Fig. 13 in Erickson 1992 we arrived at different values for both body length (0.22 m vs. 0.18 m and 0.30 m vs. 0.20 m) and tooth replacement rate (6.49 vs. 11.41 and 24.74 vs. 20.26) the later of which fall in the continuum of tooth replacement rates reported by Westergaard and Ferguson (1990). The values from Erickson (1992) are added to the other data of Erickson and this study in Fig. 11 whereas Fig. 12 does the same for the hatchling values from Erickson (1996).
We see a linear relationship of Body length and tooth replacement rate in all three variants (Fig. 10, 11, 12). The 2.5 m and 3.2 m long individuals from Erickson (1992) are most removed from the trendline in Fig. 10, but even they are not far off and the fit is relatively good ($R^2 = 0.90$). Adding the hatchling data from Erickson 1992 decreases the fit minimally ($R^2 = 0.883$) but changes the trendline by increasing the slope and lowering the intercept, so that in this version the 2.5 m individual, our 0.906 m long individual, and the smaller of the hatchlings are the most removed from the trendline (Fig. 11). Adding the hatchling data from Erickson 1996 decreases the fit even less ($R^2 = 0.893$) but still changes the trendline by increasing the slope and lowering the intercept, so that in this version the 2.5 m individual, our 0.906 m long individual, and the smaller of the hatchlings are the most removed from the trendline.

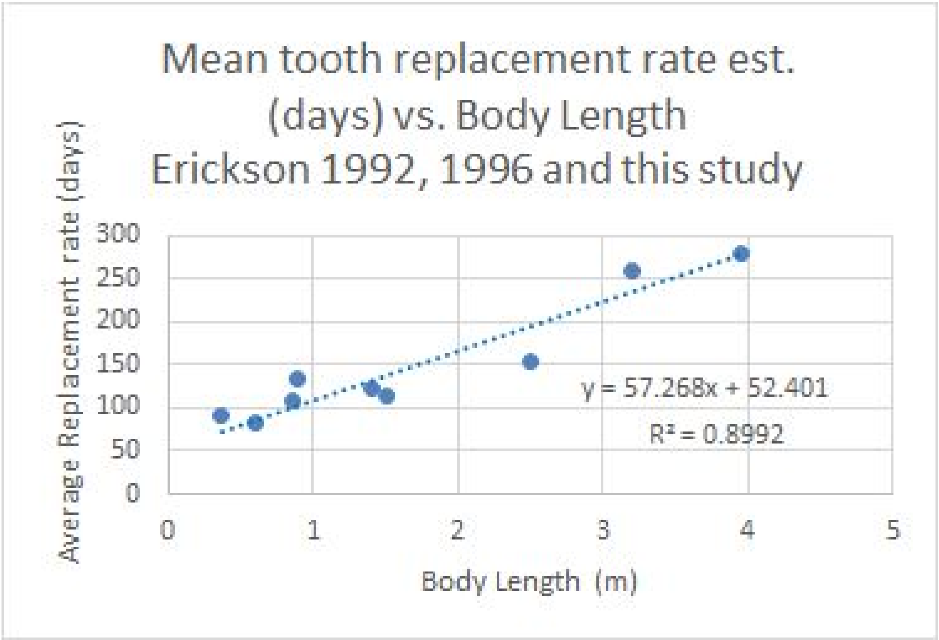

Fig. 10 Mean Tooth Replacement Rate of *Alligator* specimens by Body length
Data from Erickson (1992, 1996) was added to our three specimens to explore the relationship of tooth replacement rate and Body length. A linear function describes the relationship.

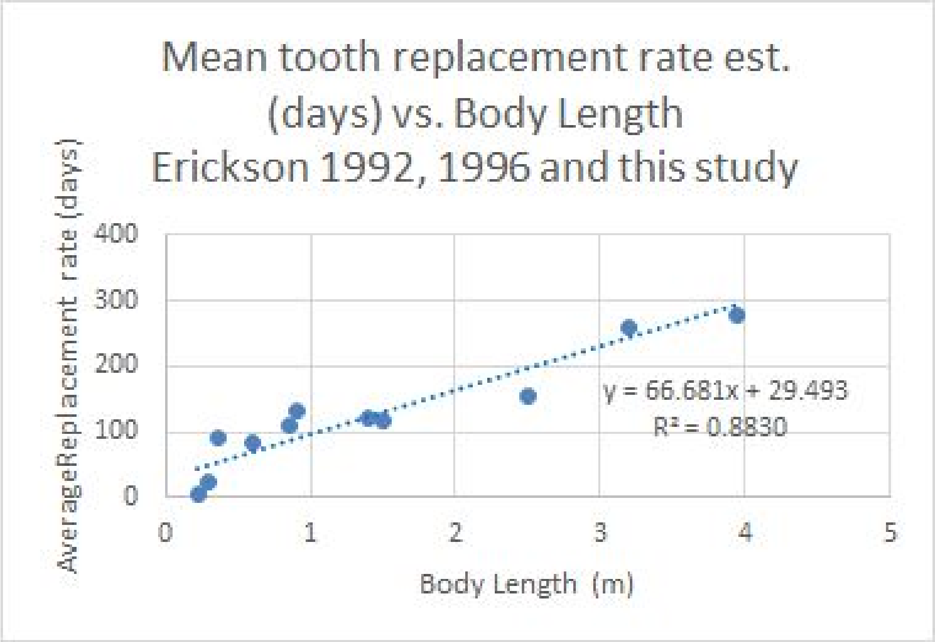

Fig. 11 Mean Tooth Replacement Rate of *Alligator* specimens by Body length with hatchling data from Erickson 1992

Data from Erickson (1992, 1996) was added to our three specimens to explore the relationship of tooth replacement rate and Body length. A linear function describes the relationship.

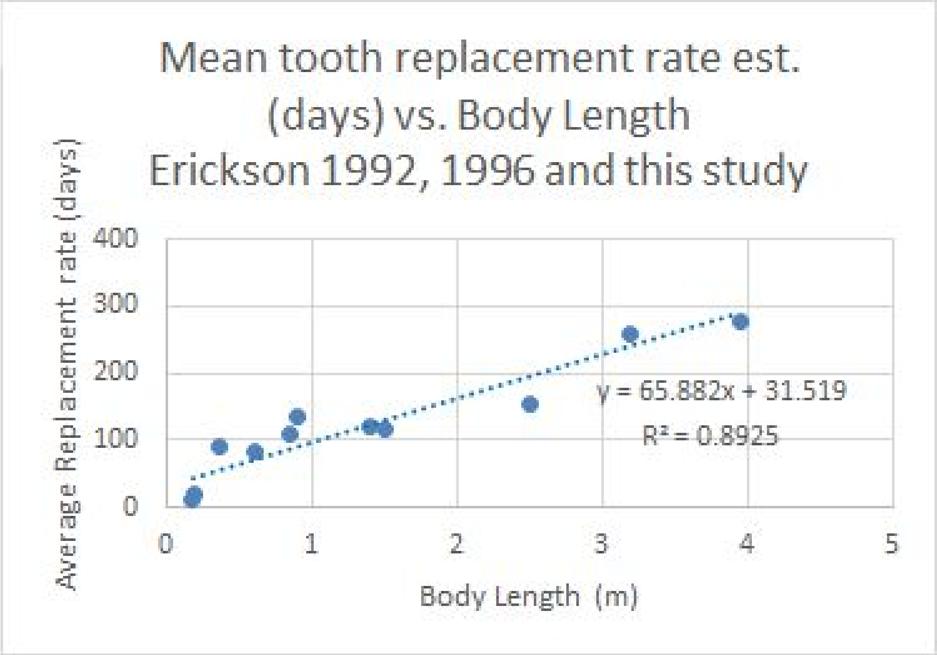

Fig. 12 Mean Tooth Replacement Rate of *Alligator* specimens by Body length with hatchling data from Erickson 1996

Data from Erickson (1992, 1996) was added to our three specimens to explore the relationship of tooth replacement rate and Body length. A linear function describes the relationship.

REFERENCES

D'Emic MD, Whitlock JA, Smith KM, Fisher DC, Wilson JA. 2013. Evolution of high tooth replacement rates in sauropod dinosaurs. *PLOS ONE* **8(7)**:e69235 DOI 10.1371/journal.pone.0069235.

Erickson GM. 1992. Verification of von Ebner incremental lines in extant and fossil archosaur dentine and tooth replacement rate assessment using counts of von Ebner lines. Master's thesis. Montana State University Bozeman, Montana.

Erickson GM. 1996. Daily deposition of dentine in juvenile Alligator and assessment of tooth replacement rates using incremental line counts. *Journal of Morphology* **228**:189–194 DOI 10.1002/(SICI)1097-4687(199605)228:2<189::AID-JMOR7>3.0.CO;2-0.

Westergaard B, Ferguson MWJ. 1990. Development of the dentition in Alligator mississippiensis: upper jaw dental and craniofacial development in embryos, hatchlings, and young juveniles, with a comparison to lower jaw development. *American Journal of Anatomy* **187(4)**:393–421 DOI 10.1002/aja.1001870407.
