# Supplementary material for: Sampling impacts the assessment of tooth growth and replacement rates in archosaurs: implications for paleontological studies
Source: PeerJ. 2020 Sep 18;8:e9918. doi: 10.7717/peerj.9918 (PMC7505082; doi:10.7717/peerj.9918)
Supplement: Supplemental Information 2 [file peerj-08-9918-s002.pdf]

crown height [mm]	0.1928	0.2028	0.9228	1.1750	1.2328	1.5628	1.6228	1.6428	1.7850	1.7928	1.8628	2.0528	2.0550
TFT by sampled CA sections [days]	16.0667	17.6348	70.9846	90.3846	70.4457	130.2333	121.7100	131.4240	89.2500	96.9081	128.4690	175.9543	205.5000
VEIW of the tooth [mm]	0.0120	0.0115	0.0130	0.0130	0.0175	0.0120	0.0133	0.0125	0.0200	0.0185	0.0145	0.0117	0.0100
Tooth specific SD [mm]	0.003	0.0025	0.0030	0.0037	0.0048	0.0030	0.0030	0.0028	0.0054	0.004	0.0030	0.0030	0.0020
TFT (tooth specific SD) + [days]	21.4222	22.5333	92.2800	126.3441	97.0709	173.6444	157.0452	169.3608	122.2603	123.6414	161.9826	236.8615	256.8750
TFT (tooth specific SD) - [days]	12.8533	14.4857	57.6750	70.3593	55.2825	104.1867	99.3551	107.3725	70.2756	79.6800	106.4457	139.9636	171.2500
SD + [days]	5.3556	4.8986	21.2954	35.9595	26.6252	43.4111	35.3352	37.9368	33.0103	26.7333	33.5136	60.9073	51.3750
SD - [days]	-3.2133	-3.1491	-13.3096	-20.0253	-15.1632	-26.0467	-22.3549	-24.0515	-18.9744	-17.2281	-22.0233	-35.9906	-34.2500
SD + [%]	33.3333	27.7778	30.0000	39.7849	37.7953	33.3333	29.0323	28.8660	36.9863	27.5862	26.0870	34.6154	25.0000
SD - [%]	-20.0000	-17.8571	-18.7500	-22.1557	-21.5247	-20.0000	-18.3673	-18.3007	-21.2598	-17.7778	-17.1429	-20.4545	-16.6667

crown height [mm]	2.0628	2.1328	2.1928	3.4750	4.5650	5.0250	5.2650	5.6550	6.2250	6.4150	8.8250	12.8650	Mean	Variance	t -statistic	p-value
TFT by sampled CA sections [days]	134.5304	148.1111	173.1158	133.6538	249.0000	301.5000	195.0000	396.8421	364.0351	342.1333	519.1176	559.3478	194.4541	20739.047		
VEIW of the tooth [mm]	0.0153	0.0144	0.0127	0.0260	0.0183	0.0167	0.0270	0.0143	0.0171	0.0188	0.0170	0.0230				
Tooth specific SD [mm]	0.0030	0.0037	0.0030	0.0080	0.0043	0.0035	0.0065	0.0080	0.0110	0.0053	0.0030	0.0050				
TFT (tooth specific SD) + [days]	167.2541	199.3271	226.8414	193.0556	325.9938	381.6456	256.8293	904.8000	1020.4918	476.9517	630.3571	714.7222	290.3837	69121.160	1.6001	0.0590
TFT (tooth specific SD) - [days]	112.5164	117.8343	139.9660	102.2059	201.4267	249.1736	157.1642	254.1573	221.5302	266.7360	441.2500	459.4643	152.5044	12859.536	-1.1443	0.1293
SD + [days]	32.7236	51.2160	53.7256	59.4017	76.9938	80.1456	61.8293	507.9579	656.4567	134.8183	111.2395	155.3744				
SD - [days]	-22.0141	-30.2769	-33.1498	-31.4480	-47.5733	-52.3264	-37.8358	-142.6848	-142.5048	-75.3974	-77.8676	-99.8835				
SD + [%]	24.3243	34.5794	31.0345	44.4444	30.9212	26.5823	31.7073	128.0000	180.3279	39.4052	21.4286	27.7778				
SD - [%]	-16.3636	-20.4420	-19.1489	-23.5294	-19.1058	-17.3554	-19.4030	-35.9551	-39.1459	-22.0374	-15.0000	-17.8571				

TFTs based on mean VEIW [tooth] plus/minus 1 SD [tooth]

TFTs based on mean VEIWs for the sampled tooth positions sorted by ascending cental axis height. The light blue rows TFT +/- SD show the TFT based on the VEIW of the respective tooth plus or minus the standard deviation of all VEIWs with a transect orientation along the CA in that particular tooth. These SDs are presented in the row tooth specific SD for each tooth. The next two rows are showing just the differences between TFTs based on mean VEIW [tooth] minus 1 SD [tooth] (TFT+ SD, as it produces bigger TFTs) or TFT and the calculated TFT in days based on men VEIW [tooth] plus 1 SD [tooth] (TFT- SD as it produces smaller TFTs) and the two rows below them are showing the same differences in percent (see below). The last four columns show the mean TFT, the variance, t-test statistic and the p-value for a one sided two sample t-test comparing the upper and lower TFT ranges based on the mean SD of tooth VEIWs. Both are not significantly different from the TFT based on sampling.

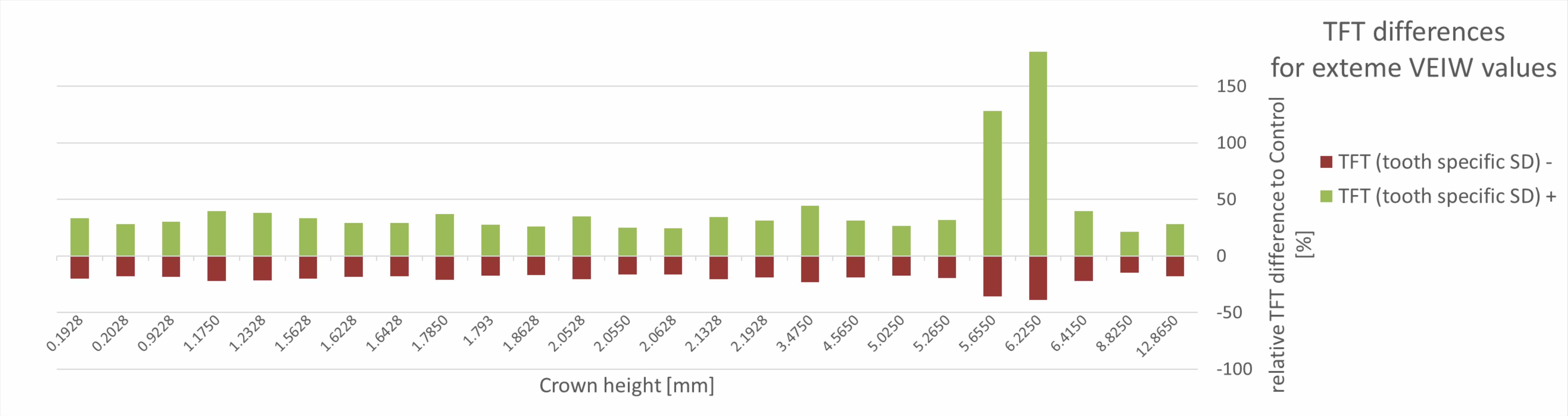

TFT differences for extreme VEIW values using tooth specific SDs

For each tooth (on the x-axis with their crown height) the deviation of the upper and lower TFT estimate (upper estimate: based on mean VEIW [tooth] minus 1 SD [tooth]; lower estimate based on mean VEIW [tooth] plus 1 SD [tooth]) to the calculated TFTs [based on mean VEIW [tooth]) is shown in percent (see last two rows of table 4). Lower TFT estimates are in red-brown, upper TFT estimates in green.
