# Supplementary material for: Sampling impacts the assessment of tooth growth and replacement rates in archosaurs: implications for paleontological studies
Source: PeerJ. 2020 Sep 18;8:e9918. doi: 10.7717/peerj.9918 (PMC7505082; doi:10.7717/peerj.9918)
Supplement: Supplemental Information 3 — Mean VEIW for the sampled tooth positions in mm. Only measurements with the same transect orientation were used (central axis, excerpt for ’ root base; * a combination of root base and crown base transects). Some tooth positions were sampled but had a different transect orientation from the other teeth and were excluded from table and analyses. Second generation replacement teeth (one in the dentition of the smallest specimen, four in the dentition of the medium sized specimen, three in the dentition of the largest specimen) are excluded. ANOVA result table for the three specimens. ANOVAs based on VEIWs of all sampled alveoli with a central axis orientation and of just those alveoli with three or more central axis samples below the table, using its values. [file peerj-08-9918-s003.doc]

|  | Tooth position | 1 | 2 | 3 | 4 | 11 | 12 | 15 | 16 | 17 | 18 | 19 |
| --- | --- | --- | --- | --- | --- | --- | --- | --- | --- | --- | --- | --- |
| Small Alligator  NCSM 100803 | functional teeth upper dentition |  |  | 0.019 |  | 0.0127 | 0.0153 |  |  | 0.0120 | 0.0117 |  |
|  | replacement teeth upper dentition |  |  |  |  |  |  |  |  | 0.0115 |  |  |
|  | functional teeth lower dentition |  | 0.0133 |  | 0.0175 |  | 0.0144 |  |  |  | 0.0145 | 0.0125 |
|  | replacement teeth lower dentition |  |  |  | 0.0120 |  | 0.0130 |  |  |  |  |  |
| Medium Alligator  NCSM 100804 | functional teeth upper dentition |  |  | 0.0156* |  | 0.0115* |  |  |  | 0.009' | 0.0135' | 0.0094* |
|  | replacement teeth upper dentition |  |  | 0.0246 |  | 0.0370 |  |  |  |  | 0.0153 | 0.0240 |
|  | functional teeth lower dentition |  |  |  |  | 0.0115* | 0.0143* |  |  |  | 0.0128* |  |
|  | replacement teeth lower dentition |  |  |  |  | 0.0230 |  |  |  |  |  |  |
| Large Alligator  NCSM 100805 | functional teeth upper dentition | 0.0143 |  |  | 0.0230 | 0.0170 | 0.0171 |  |  |  | 0.0188 |  |
|  | replacement teeth upper dentition |  | 0.0130 |  | 0.0183 | 0.0100 | 0.0200 |  |  |  |  |  |
|  | functional teeth lower dentition |  |  |  |  | 0.0167 |  |  | 0.0270 |  |  |  |
|  | replacement teeth upper dentition |  |  |  |  |  |  | 0.0260 |  |  |  |  |

| ANOVA for the three specimens | |  | | |  |  |
| --- | --- | --- | --- | --- | --- | --- |
|  | SS | df | MS | F | F crit | p |
| Between Groups | 0.000145 | 2 | 7.24235E-05 | 2.2954 | 3.27 | 0.1157 |
| Within Groups | 0.001104 | 35 | 3.15519E-05 |  | From F table for  alpha = 0.05 |  |
| total | 0.001249 | 37 |  |  |
|  |  |  |  |  |  |  |
|  | overall | small | medium | large |  |  |
| Mean VEIW [mm] | 0.01635 | 0.01376 | 0.01703 | 0.01843 |  |  |
| SD | 0.00581 | 0.00222 | 0.00799 | 0.00504 |  |  |

| ANOVA for all sampled alveoli in CA orientation | | | |  |  |  |
| --- | --- | --- | --- | --- | --- | --- |
|  | SS | df | MS | F | F crit | p |
| Between Groups | 0.000367 | 10 | 3.67307E-05 | 1.0417 | 2.38 | 0.4481 |
| Within Groups | 0.000670 | 19 | 3.5261E-05 |  | From F table for alpha = 0.05 |  |
| total | 0.001037 |  |  |  |
|  |  |  |  |  |  |  |
| ANOVA for alveoli with 3 or more samples (4, 11, 12, 18) | | | |  |  |  |
|  | SS | df | MS | F | F crit | p |
| Between Groups | 5.67124E-05 | 3 | 1.89041E-05 | 0.4844 | 3.29 | 0.6981 |
| Within Groups | 0.000585 | 15 | 3.90235E-05 |  | From F table for alpha = 0.05 |  |
| total | 0.000642 |  |  |  |

**Supplemental table S–3**

Mean VEIW for the sampled tooth positions in mm. Only measurements with the same transect orientation were used (central axis, excerpt for ' root base; * a combination of root base and crown base transects). Some tooth positions were sampled but had a different transect orientation from the other teeth and were excluded from table and analyses. Second generation replacement teeth (one in the dentition of the smallest specimen, four in the dentition of the medium sized specimen, three in the dentition of the largest specimen) are excluded. ANOVA result table for the three specimens. ANOVAs based on VEIWs of all sampled alveoli with a central axis orientation and of just those alveoli with three or more central axis samples below the table, using its values.
